# Scaffolded AI-Supported Problem-Based Learning for Medical Interns: Exploratory Retrospectively Registered Randomized Controlled Evaluation With a Voluntary Feasibility Follow-Up

**DOI:** 10.2196/95039

**Published:** 2026-09-03

**Authors:** Zhengqi Zhao, Baijing Wu, Shuyuan Tian, Haigen Hu, Pengyi Hao

**Affiliations:** 1 Department of Computer Science and Technology, Zhejiang University of Technology, No. 288 Liuhe Road, Xihu District, Hangzhou, Zhejiang, 310023, China, 86 158 6913 2329; 2 Zhejiang Institute of Artificial Intelligence, Hangzhou, Zhejiang, China; 3 Department of Ultrasound, Tongde Hospital Affiliated to Zhejiang Chinese Medical University, No. 234 Gucui Road, Hangzhou, Zhejiang, China

**Keywords:** problem-based learning, clinical reasoning, scaffolding, artificial intelligence, large language models, educational technology, randomized controlled trial

## Abstract

**Background:**

High-quality problem-based learning (PBL) during internship is resource-intensive and difficult to scale without consistent facilitation. Although generative AI is increasingly used in health professions education, many applications remain on-demand answer tools that may not reproduce core PBL processes.

**Objective:**

This study aimed to develop and evaluate the Multiagent PBL Environment for Clinical Reasoning (MAPLE-CR), an AI-supported environment that uses generative AI as a process-oriented scaffold rather than an answer-delivery aid. The system supports cognitive mechanisms through reasoning prompts, social-interactional mechanisms through simulated tutor and peer roles, and regulatory mechanisms through structured workflows and feedback loops. We separately evaluated implementation and repeated-use feasibility, including learner experience, short-term outcomes versus self-study, and preliminary framework-aligned process evidence.

**Methods:**

We conducted a 2-stage study. In an exploratory randomized evaluation (N=52; intervention: n=26; control: n=26), interns completed parallel pretests and posttests around a standardized case. The intervention group engaged in asynchronous, scaffolded PBL in MAPLE-CR, whereas controls completed case-matched self-study using materials derived from the same case and learning objectives. We summarized transcript-derived process dimensions and postsession learner experience in the intervention group. In a voluntary follow-up, 18 participants completed 1 MAPLE-CR case per week for 4 additional weeks to examine repeated use feasibility and patterns across novel cases.

**Results:**

Baseline pretest scores were comparable between groups (*P*=.56). The intervention group achieved higher posttest scores than controls (Hodges-Lehmann median difference 8.25 points, 95% CI 6.60-11.60; Cliff δ=0.506, 95% CI 0.21‐0.76; *P*=.001) and greater score gains (median difference 6.60 points, 95% CI 1.60-14.95; *P*=.01). On a 0 to 5 scale, mean scores were highest for knowledge accuracy (4.4) and clinical reasoning (CR, 3.7), whereas active participation averaged 2.1, and interaction-oriented dimensions were more variable. Means for 12 positively worded questionnaire items ranged from 4.19 to 4.62, supporting high satisfaction, involvement, perceived support, and self-efficacy; open-ended responses contextualized perceived strengths and improvement needs. In the voluntary follow-up (n=18), weekly cross-case use was feasible; median within-session gains ranged from 13.4 to 20.0 points across 5 attempts, with marked increases in CR and aggregate scores from attempts 1 to 2 but statistically uncertain later trajectories.

**Conclusions:**

MAPLE-CR was feasible and showed high learner acceptability. Its use was associated with larger short-term CR knowledge gains than case-matched self-study, while transcript analyses provided preliminary framework-aligned process evidence. However, the post hoc sensitivity analysis did not establish prospective power sufficiency, and the confidence interval could not exclude smaller educationally meaningful effects. These findings suggest that AI-supported, process-oriented PBL may provide a scalable formative supplement for CR practice when facilitator capacity and small-group scheduling are constrained, but the study did not isolate specific scaffolding effects. Further research should confirm these findings using larger, prospectively powered trials with stronger active comparators and longer-term outcome measures.

## Introduction

Medical interns must translate biomedical knowledge into timely clinical decisions under uncertainty. Problem-based learning (PBL) supports this transition by engaging learners in case analysis, hypothesis generation, interpretation of evolving information, and iterative revision of explanations and differential diagnoses [1,2]. Its educational value depends not only on exposure to cases but also on learners’ active articulation of reasoning, identification of knowledge gaps, self-directed study, and integration of new understanding [3-5].

Despite these benefits, scaling high-quality PBL in routine clinical curricula remains difficult. Effective PBL typically depends on skilled facilitation, small-group coordination, and protected instructional time, all of which are resource-intensive and difficult to sustain consistently during internship [6]. In practice, the challenge is not only increasing access to PBL-like activities but also preserving the pedagogical processes that make PBL distinctive. Its educational value depends on collaborative sensemaking, externalization of reasoning, and facilitator-guided progression through a structured case process [7]. When these enabling processes are weak or absent, PBL can deteriorate into superficial discussion or premature answer-seeking rather than disciplined reasoning.

Against these implementation constraints, generative AI, especially large language model (LLM) systems capable of flexible multiturn dialogue, has emerged as a potential way to expand learning opportunities in time- and resource-constrained clinical settings [8-12]. Early work in health professions education has explored generative AI for functions such as content support, explanation, feedback, and learner assistance [13-15]. Several studies have also begun to examine AI-supported learning in PBL-related contexts, with encouraging findings for learner support, idea generation, and just-in-time explanation [15,16]. However, in many of these implementations, AI functions primarily as a consultation aid or question-answering assistant embedded within the learning process, rather than as support for orchestrating the PBL process itself.

This distinction matters because question answering is not equivalent to PBL. PBL is not only a matter of information access [1,17]; it is a structured learning process in which learners are expected to generate and justify hypotheses, respond to new information, engage with alternative perspectives, and move through recognizable phases of inquiry [18]. Its benefits also depend on feedback and continuity across cases, so that learners can reflect on performance, recalibrate reasoning, and improve over time [19]. Accordingly, simply providing answers or ad hoc explanations is unlikely to reproduce the core pedagogical functions of PBL. The specific gap addressed in this study is therefore not the absence of AI assistance within case learning but the limited evidence on AI-supported PBL environments designed to scaffold multiple phases of the PBL cycle as a facilitator-like process [20], integrating presession preparation, in-session reasoning and peer interaction, postsession feedback, and subsequent practice rather than supporting only isolated question answering or explanation.

In this study, we adopt a process-oriented view of scaffolding for AI-supported PBL. We use scaffolding to mean structured support embedded in the learning workflow to help learners perform reasoning, interaction, and reflection activities that would otherwise require sustained tutor facilitation. We use orchestration to refer to the coordination of scaffolded activities across the full PBL cycle, including presession preparation, case discussion, postsession feedback, and subsequent practice. Within this theoretical framework, MAPLE-CR (Multiagent PBL Environment for Clinical Reasoning) was designed around 3 complementary forms of process-oriented scaffolding. First, cognitive scaffolding prompts learners to generate hypotheses, justify them with case evidence, revise interpretations as new information emerges, and synthesize key clinical points [1,5,17]. Second, social-interactional scaffolding uses simulated tutor and peer roles to sustain dialogue, questioning, turn-taking, peer-responsive reasoning, and externalization of thinking rather than a purely monologic learner-AI exchange [21-23]. Third, regulatory scaffolding structures the workflow, monitors objective coverage, manages phase transitions, and embeds feedback loops across repeated sessions to support reflection, calibration, and progressive improvement [5,19,24]. This framework treats scalability not simply as replacing human labor with automation but as preserving the cognitive, dialogic, and feedback-regulatory functions that make PBL educationally meaningful.

Guided by this framework, we developed MAPLE-CR, an AI-supported learning environment for continuous, scaffolded PBL practice among medical interns. It was intended to provide a structured yet flexible environment for clinical reasoning (CR) practice when in-person facilitation and repeated small-group sessions are difficult to sustain. Accordingly, this study aimed to evaluate MAPLE-CR across three distinct evidentiary domains: (1) feasibility of implementation and repeated cross-case use, together with learner acceptability and perceived educational value; (2) short-term learning-outcome differences relative to case-matched self-study in an exploratory randomized evaluation; and (3) preliminary framework-aligned process evidence based on transcript-derived formative dimensions, with expert ratings used as an external reference (Table 1 summarizes this mapping and related expert-rating checks). The voluntary follow-up was interpreted descriptively as evidence of repeated-use feasibility rather than longitudinal learning effectiveness.

**Table 1.** Intended mapping of Multiagent Problem-based Learning Environment for Clinical Reasoning (MAPLE-CR) components to scaffolding functions, formative dimensions, and expert-rating validation.

Scaffolding function	MAPLE-CR components and intended PBL^a^ support	Framework-aligned formative dimensions and expert-rating validation
Cognitive scaffolding	Learning objectives, sequential case scenarios, and tutor prompts for hypothesis generation, evidence justification, revision, and synthesis. These components were intended to elicit diagnostic and management reasoning aligned with case objectives.	CR^b^; KA^c^; expert ratings for CR and KA
Social-interactional scaffolding	Tutor and peer roles, peer prompts and counterpoints, and turn-taking support. These components were intended to sustain multivoice discussion and externalization of reasoning.	AP^d^; CC^e^; expert ratings for AP and CC
Regulatory scaffolding	Case recommendation, pretest-informed learner profile, objective-coverage monitoring, phase transitions, summaries, feedback, and updated learning record. These components were intended to structure reflection, calibration, and repeated practice.	SR^f^; expert ratings for SR

a PBL: problem-based learning.

b CR: clinical reasoning.

c KA: knowledge accuracy.

d AP: active participation.

e CC: communication and collaboration.

f SR: summarization and reflection.

## Methods

### MAPLE-CR

**Figure 1.**
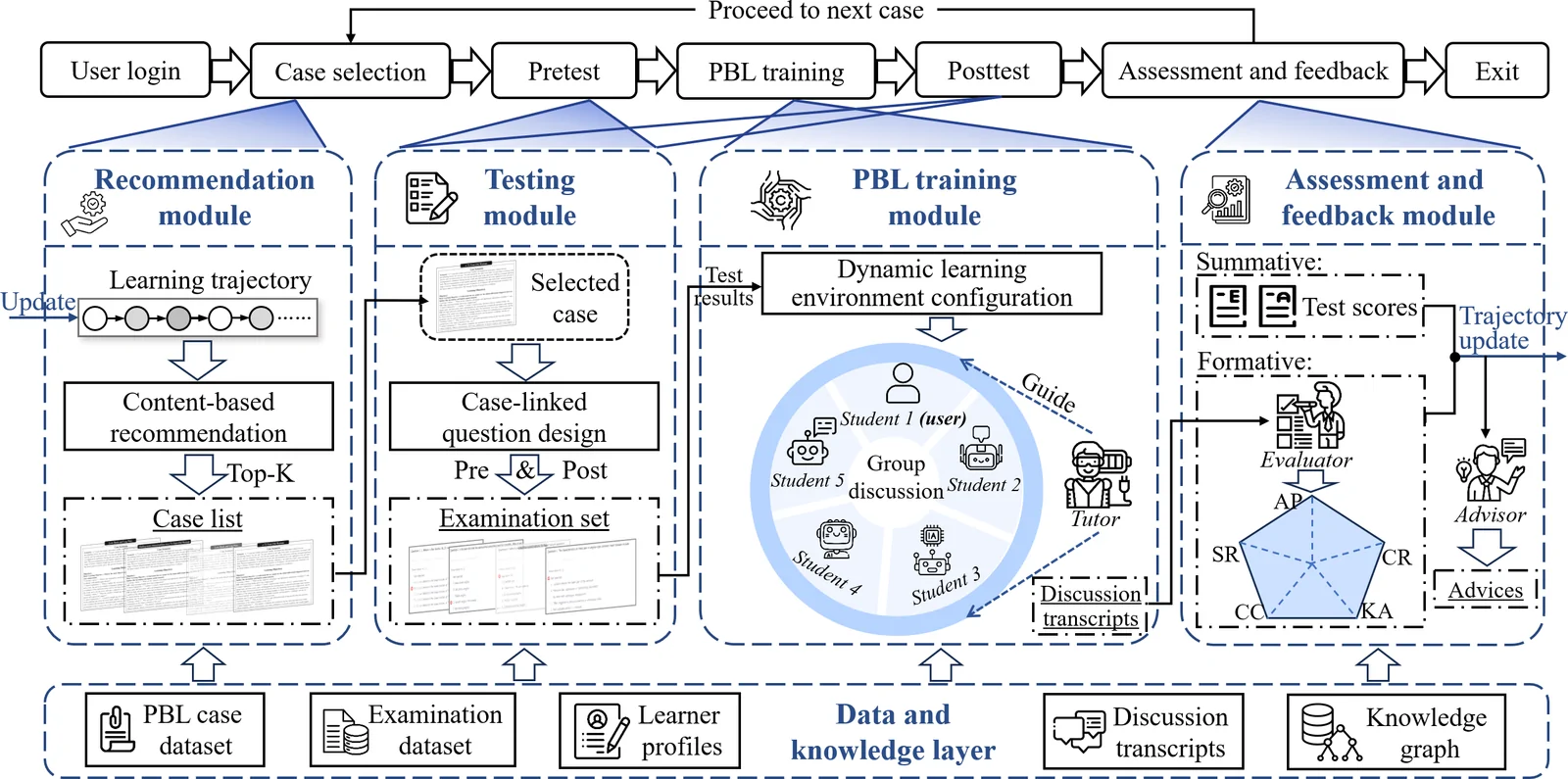
Multiagent Problem-based Learning Environment for Clinical Reasoning closed-loop problem-based learning workflow. Learners select a recommended case, complete a brief pretest, engage in scenario-based small-group discussion with a tutor role and simulated peers, and then complete a posttest. Transcript-based formative feedback is generated across 5 dimensions. Session outputs update the learner’s trajectory to inform subsequent case recommendations, enabling repeated, scaffolded practice. AP: active participation; CC: communication and collaboration; CR: clinical reasoning; KA: knowledge accuracy; PBL: problem-based learning; SR: summarization and reflection.

MAPLE-CR is a web-based, LLM-supported PBL environment for medical interns; an overview of the system structure and learning workflow is shown in Figure 1. In implementation terms, an “agent” refers to an LLM-guided software role rather than a human participant, consistent with recent work on LLM-based agents and multiagent collaboration [25,26]. MAPLE-CR instantiates tutor, peer, evaluator, and advisor functions as distinct agents and uses multiagent orchestration to coordinate role interactions and workflow sequencing. Learners interact with MAPLE-CR through 4 connected modules: recommendation, testing, PBL training, and assessment with feedback, all supported by a shared data and knowledge layer containing cases, learning objectives, examination items, learner records, discussion transcripts, formative scores, and knowledge graph resources.

The recommendation and testing modules initiate each learning cycle. The recommendation module uses the learner’s prior learning trajectory, defined as the longitudinal record of completed cases and assessment history, to generate a ranked list of candidate cases. The learner selects 1 recommended case, preserving learner choice while supporting continuity across repeated practice. The testing module then administers brief case-linked pretests and posttests aligned with the case learning objectives and target knowledge points; the pretest summarizes baseline case-relevant understanding, whereas the posttest assesses immediate knowledge performance after the PBL session.

The PBL training module is the objective-guided learning environment. Cases are organized around prespecified learning objectives and sequential scenarios. Within each scenario, the tutor agent presents case information, monitors objective coverage, and supports transition to summary phases. Peer-agent personas combine fixed role constraints with learner-specific ability calibration: the fixed prompt defines the peer as a medical learner who participates in discussion, asks questions, and responds to others, whereas the dynamic component adjusts the peer agent’s ability profile according to the learner’s pretest-derived baseline.

The assessment with feedback module combines summative and formative information. Summative feedback includes the pretest score, posttest score, and score change. Formative assessment is generated from the discussion transcript across 5 PBL-relevant dimensions aligned with the scaffolding framework: active participation (AP), CR, knowledge accuracy (KA), communication and collaboration (CC), and summarization and reflection (SR). AP is computed algorithmically from learner interaction frequency, whereas CR, KA, CC, and SR are scored by an evaluator agent using dimension-specific prompts and the full discussion context. CR and KA additionally use retrieval from the structured medical knowledge graph as a clinical reference [14]. Evaluator-agent scores were constrained to a 0 to 5 scale in 0.5-point increments and accompanied by brief dimension-specific justifications. The resulting feedback summarizes strengths and areas for improvement and updates the learner record for subsequent case recommendations.

**Figure 2.**
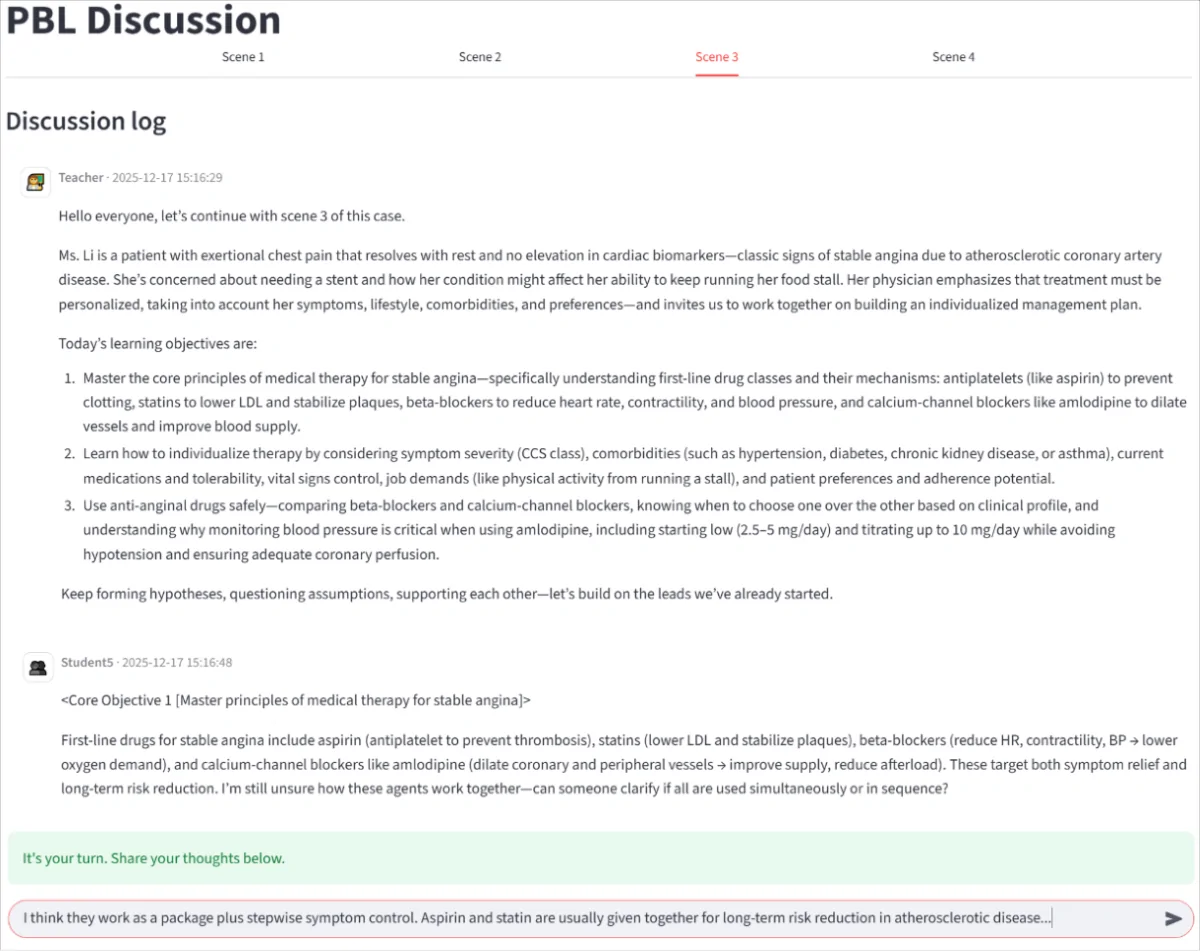
User interface of the problem-based learning (PBL) training module in Multiagent PBL Environment for Clinical Reasoning. The screenshot shows a later management-oriented scenario in which the tutor agent presents staged case information and learning objectives to structure discussion around the selected case. Within this objective-guided format, the peer agent initiates discussion and the learner contributes through text input. BP: blood pressure; HR: heart rate; LDL: low-density lipoprotein.

MAPLE-CR was implemented as a remotely accessed web platform, with a Streamlit interface deployed on a cloud server. Model inference used the Qwen-flash API (qwen-flash-2025-07-28) [27], with temperature set to 0.7. Only pseudonymized educational content was transmitted to the Qwen API for model inference; direct personal identifiers, account credentials, and study-specific participant identifiers were excluded from API requests. The user interface for the training module is shown in Figure 2. Further details on API data flow, access control, data retention, and consent are provided in Multimedia Appendix 1. Multimedia Appendix 2 provides a video demonstration of the system, and Multimedia Appendix 1 provides additional implementation details, including module interactions, system configuration, peer-agent calibration, and agent prompts.

### Study Design

We conducted an exploratory randomized controlled educational evaluation followed by a voluntary 4-week repeated-use feasibility follow-up. Reporting was guided by the CONSORT-eHEALTH (Consolidated Standards of Reporting Trials of Electronic and Mobile Health Applications and Online Telehealth) checklist (Checklist 1).

Fifty-two first-year postgraduate medical interns (resident interns) were recruited from Tongde Hospital Affiliated to Zhejiang Chinese Medical University, a tertiary teaching hospital in Zhejiang Province, China. All had prior face-to-face PBL experience and reported previous use of GPT-style chatbots, but none had used MAPLE-CR. Participants were randomized 1:1 to the intervention or control group using a computer-generated sequence prepared by a researcher not otherwise involved in the trial. Participant recruitment and enrollment took place from December 6 to 15, 2025, and the randomized educational evaluation was conducted on December 19, 2025, before trial registration; therefore, the trial was retrospectively registered.

MAPLE-CR system and code development, including backend model integration and platform deployment, began in August 2025 and was followed by internal testing and debugging by the research team before participant recruitment. These deployment and development activities were limited to internal testing and debugging and did not involve participant enrollment, randomization, or collection of trial outcome data.

All participants completed the pretest concurrently in an on-site computer laboratory. To reduce contamination, groups were seated in separate classrooms. Research team members monitored the classrooms to maintain timing and ensure access to the assigned materials or MAPLE-CR platform; they did not provide clinical teaching, facilitate discussion, answer case-related questions, or collect additional outcome data beyond documenting attendance and session completion. Allocation was concealed until completion of the pretest; participant blinding after allocation was not feasible. Pretest, posttest, questionnaire, and follow-up records were linked using study-specific participant identifiers; analyses were conducted on deidentified datasets without names or other direct personal identifiers. To standardize exposure, all participants used the same standardized PBL case, and the recommendation function was therefore not used in the randomized component. The case focused on CR for a right middle-lobe lung mass ultimately diagnosed as lung adenocarcinoma. The case materials, learning objectives, and associated pretest and posttest items were developed and reviewed by 3 domain experts. Pretest and posttest were parallel forms based on a shared blueprint (see Multimedia Appendix 3); experts reviewed item difficulty, learning-objective coverage, and consistency between the case materials and assessment items, resolving discrepancies by consensus. Each form comprised 30 single-best-answer multiple-choice items scored automatically against a fixed answer key as the percentage of correct responses (range 0‐100), without using group-assignment information; all items had to be completed before submission. Blueprint development and expert review preceded the trial. To provide supplementary evidence for the assessment instruments, we conducted post hoc psychometric checks using actual item-level responses and independent 5-point expert difficulty ratings for all pretest and posttest items. These analyses supported acceptable internal consistency, item discrimination, and comparable expert-rated difficulty between forms; detailed methods and results are provided in Multimedia Appendix 3.

Immediately after the pretest, participants in the intervention group received a standardized 40-minute orientation to MAPLE-CR before individually completing up to 2 hours of PBL training. The orientation introduced the platform workflow, interface operations, tutor and peer roles, and basic expectations for participating in the AI-supported PBL discussion. It did not include case-specific diagnostic or management instruction, posttest answers, or substantive teaching on the target case. Because the orientation was intended to support learners’ engagement with the platform, it was considered part of the MAPLE-CR intervention package. If discussion ended early, participants reviewed the transcript until the session ended. A 2-hour case-matched self-study condition was selected as a feasible independent-learning comparator for this exploratory evaluation, using expert-reviewed materials to standardize case content and learning objectives without matching MAPLE-CR’s interaction, feedback, novelty, or procedural guidance. Both groups then completed the posttest concurrently. Because the 40-minute orientation had no time-matched counterpart in the control condition, the conditions were not equivalent in total structured exposure. Accordingly, the comparison estimates the effect of the integrated MAPLE-CR intervention package under the present implementation rather than the isolated effect of its AI-PBL scaffolding components. In the intervention group, MAPLE-CR generated transcript-based formative feedback only after the posttest; no comparable feedback was provided to controls.

After the session, participants completed a postsession questionnaire adapted from a previously published simulation-based training quality assurance instrument [28,29]. Because the original instrument was designed for simulation-based training rather than AI-supported PBL, item wording and domains were modified to fit the MAPLE-CR context. Items were rated on a 5-point Likert scale from strongly disagree to strongly agree.

Following the controlled study, intervention participants were invited to join a voluntary 4-week follow-up, completing 1 MAPLE-CR case per week with the full closed-loop workflow (recommendation, testing, PBL training, assessment, and feedback). At follow-up completion, participants provided open-ended feedback on their repeated-use experiences. The voluntary follow-up was conducted from December 22, 2025, to January 19, 2026.

No important changes to study procedures, intervention content, or platform functionality occurred after trial commencement, and no major technical failures or downtime affecting study delivery were observed.

### Sample Size and Power Considerations

No formal a priori sample size calculation was conducted. The available sample size was determined by the fixed intern cohort, the number of eligible participants who could be recruited, and the feasibility of conducting a synchronized on-site educational experiment. Accordingly, the randomized component should be interpreted as an exploratory proof-of-concept evaluation rather than a fully powered confirmatory efficacy trial. To clarify the detectable magnitude under the available sample, we conducted a post hoc sample size sensitivity analysis based on the observed posttest effect. The observed Cliff δ was 0.506, corresponding to an area under the curve of (δ+1)/2=0.753 and an approximate Cohen *d* of 0.97. With 26 participants per group and 2-sided α=.05, this observed effect was larger than the standardized mean difference corresponding approximately to 80% power (*d*≈0.78) and 90% power (*d*≈0.90). However, the lower bound of the 95% CI for Cliff δ (0.21) corresponds to an approximate *d* of 0.38, indicating that the effect-size estimate remains imprecise and that smaller but educationally meaningful effects cannot be excluded. This analysis is therefore presented only as a descriptive sensitivity analysis and does not substitute for a prospective sample size calculation.

### Registration and Analysis Status

The registration was completed retrospectively and specified broad outcome domains related to CR ability and knowledge mastery; it did not prospectively specify the operational outcomes, hypotheses, statistical analysis plan, or voluntary follow-up measures used in this educational evaluation. The pretest and posttest knowledge assessments, postsession questionnaire, transcript-based formative assessment, and voluntary repeated-use follow-up were incorporated into the study procedures; however, because registration followed the randomized evaluation, none constituted a prospectively registered confirmatory outcome, and their analyses are interpreted as exploratory or descriptive. Expert-rating convergence, session-level correlations, post hoc psychometric checks, the questionnaire response-pattern sensitivity analysis, and sample size sensitivity calculations were not specified in the registration and are reported as exploratory, sensitivity, or post hoc analyses, as applicable.

### Statistical Analyses

All 52 randomized participants completed the pretest and posttest and were analyzed as assigned under an intention-to-treat approach; intervention questionnaires and transcripts were complete, and no imputation was required. We used primarily nonparametric methods because of the modest sample size and the ordinal nature of questionnaire data. Baseline group characteristics and between-group outcomes (pretest, posttest, and score gain) were compared using Fisher exact tests or Mann-Whitney *U* tests, as appropriate. Within-group pretest-posttest changes were assessed using Wilcoxon signed-rank tests. For repeated-use outcomes across 5 attempts, we used Friedman tests for repeated within-subject comparisons. We report effect sizes and 95% CIs where applicable (eg, Cliff δ, Hodges-Lehmann median differences, or bootstrap CIs). All tests were 2-sided with α=.05. Among intervention participants only, transcript-based formative scores were summarized for the 26 MAPLE-CR sessions. Convergent validity was examined using Spearman rank correlations (ρ) and mean absolute error (MAE) against mean expert ratings, and exploratory session-level associations within the intervention group were examined using 2-sided Spearman rank correlations (ρ), given the small sample size and the bounded or ordinal nature of several variables. Because no formal a priori sample size calculation was conducted, we supplemented the randomized outcome analysis with a post hoc sample size sensitivity analysis based on the observed posttest Cliff δ, converting Cliff δ to area under the curve and to an approximate standardized mean difference. Interrater reliability of the expert reference standard was assessed for each PBL dimension using a 2-way mixed-effects absolute-agreement intraclass correlation coefficient (ICC). Because convergent-validity analyses used mean expert ratings, we report ICC(A,3), the reliability of the 3-rater mean.

### Ethical Considerations

The study was conducted in accordance with the Declaration of Helsinki and approved by the Ethics Committee of Tongde Hospital of Zhejiang Province (KTSC2025080). The trial was retrospectively registered with the Chinese Clinical Trial Registry (ChiCTR2500115799; registered on December 31, 2025). Participation was voluntary, and all participants provided informed consent before study participation, including consent to the cloud-based AI processing and research use of pseudonymized dialogue, learning, and assessment data. Data were deidentified before analysis. This was a minimal-risk educational study using simulated cases.

## Results

### Results of the Randomized Controlled Study

**Figure 3.**
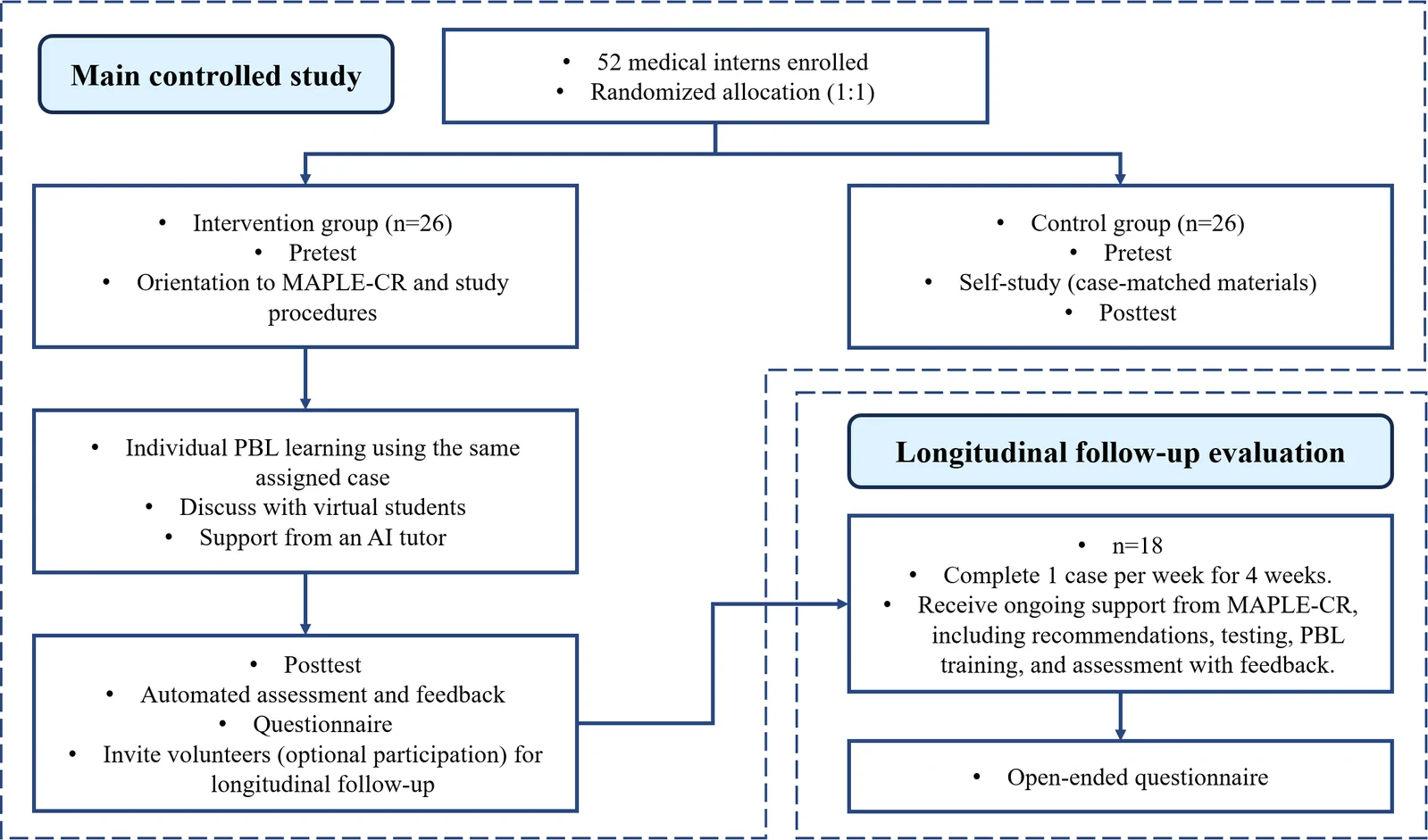
Study design and participant flow through the randomized evaluation and voluntary feasibility follow-up. Fifty-two interns were randomized 1:1 to Multiagent Problem-based Learning Environment for Clinical Reasoning (MAPLE-CR) or case-matched self-study and were included in the randomized analysis. A voluntary subset of MAPLE-CR participants subsequently completed repeated follow-up sessions across additional cases. PBL: problem-based learning.

Fifty-two interns were randomized (intervention: n=26, control: n=26), and 18 completed the voluntary follow-up (Figure 3). Baseline sex and age were comparable between the intervention and control groups (female/male: 18/8 vs 20/6, Fisher exact test, *P*=.76; median age 23, IQR 23-24 vs 24, IQR 23-24, Mann-Whitney *U* test, *P*=.31). The follow-up subgroup did not differ from the intervention group in sex (female/male: 11/7 vs 18/8, Fisher exact test *P*=.75) or age (median 23, IQR 23-24 vs 23, IQR 23-24, Mann-Whitney *U* test, *P*=.78).

**Figure 4.**
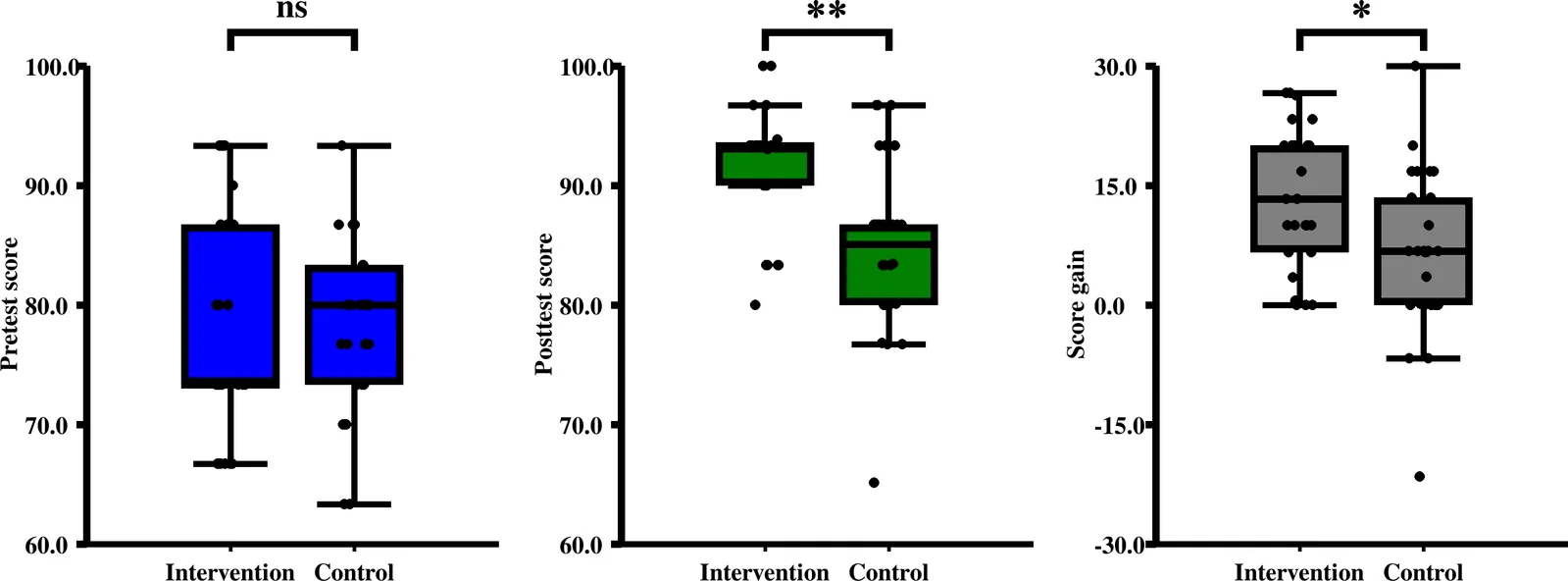
Group distributions of pretest scores, posttest scores, and score gain. Scores are on a 0 to 100 scale. Data were analyzed using the Mann-Whitney *U* test. *P<.05. **P<.01. ns: not significant.

Baseline pretest scores were comparable between the intervention and control groups (Mann-Whitney *U* test, *P*=.56; Figure 4), suggesting no meaningful baseline imbalance in initial knowledge. After the session, the intervention group achieved higher posttest scores than the control group (Mann-Whitney *U* test, *P*=.001), with a between-group median difference of 8.25 (95% CI 6.60‐11.60) points. The corresponding effect size was Cliff δ=0.506 (95% CI 0.21-0.76), indicating a distributional shift toward higher posttest scores in the intervention group. Consistently, the intervention group demonstrated a greater score gain (posttest minus pretest) than the control group (Mann-Whitney *U* test, *P=*.01), with a between-group median difference of 6.60 (95% CI 1.60‐14.95) points. The CI for this gain difference was wide, indicating uncertainty in the magnitude of the incremental benefit. Within-group analyses indicated that the control group also improved from pretest to posttest (Wilcoxon signed-rank test, *P=*.006), with a median gain of +6.70 (95% CI 0.10-10.05) points. This pattern is consistent with a measurable learning effect under the self-study condition rather than mere test-retest fluctuation. Overall, between-group separation was observed for posttest performance and gain under the present comparator condition. Because the control group also improved, this difference should be interpreted as a MAPLE-CR-associated short-term difference rather than evidence that the effect was attributable specifically to PBL scaffolding mechanisms.

The observed posttest effect was large in this sample. In the sample size sensitivity analysis, the observed effect magnitude exceeded the approximate large-effect threshold detectable with 26 participants per group under 2-sided α=.05. However, the confidence interval around Cliff δ was wide, and its lower bound corresponded to a substantially smaller standardized effect. The current sample therefore cannot exclude smaller but still educationally meaningful effects. Because this was a descriptive sensitivity analysis, these findings should be interpreted as evidence of a large short-term difference under the present study conditions, rather than as definitive efficacy evidence.

Table 2 summarizes postsession questionnaire responses following the MAPLE-CR intervention, presenting full-sample results as the main descriptive analysis and filtered results as a sensitivity analysis. Across the 12 positively worded items (Q1-Q12), full-sample ratings were consistently favorable, with means ranging from 4.19 to 4.62, indicating high satisfaction, sustained involvement, strong perceived support, and enhanced self-efficacy. Because Q13-Q15 were reverse-keyed, we conducted a nonprespecified sensitivity analysis excluding 5 questionnaires with all 15 ratings ≥3 to examine possible acquiescent responding; this criterion did not define invalid responses, and full-sample results remained primary. Filtered means changed minimally for Q1-Q11 (absolute mean difference ≤0.17; *|d|*≤0.23). In contrast, the 3 perceived cognitive-load-related items (Q13-Q15), reflecting perceived mental effort, perceived information overload, and interface-related distraction, showed lower means, greater dispersion, and larger shifts after filtering (mean difference=−0.31 to −0.54; *d*=−0.27 to −0.53), suggesting greater heterogeneity in perceived load and distraction. Overall, the questionnaire results support strong acceptability and perceived educational value of MAPLE-CR; however, perceived cognitive-load-related items remained salient even after filtering. This pattern highlights a broader consideration for AI-supported education: in PBL-like settings, AI may add value less by reducing cognitive demands and more by helping to orchestrate complex discourse and make reasoning visible, while minimizing avoidable interface or coordination demands that can divert effort from learning.

**Table 2.** Per-item questionnaire responses (5-point Likert scale) in the full sample (n=26) and filtered sample (n=21)^a^.

Question	Full sample, mean (variance)	Filtered, mean (variance)	Mean difference	Cohen *d*
Learning satisfaction				
Q1. I was satisfied with the overall PBL^b^ system experience.	4.19 (0.617)	4.10 (0.658)	−0.10	−0.12
Q2. The recommended cases matched my learning needs.	4.19 (0.386)	4.14 (0.408)	−0.05	−0.08
Q3. The system helped me learn the clinical reasoning for this case.	4.27 (0.581)	4.10 (0.562)	−0.17	−0.23
Q4. The test questions effectively assessed my knowledge.	4.19 (0.386)	4.10 (0.372)	−0.10	−0.16
Q5. The feedback was accurate and helpful.	4.62 (0.390)	4.57 (0.435)	−0.04	−0.07
Sense of involvement				
Q6. I stayed engaged and actively participated in reasoning/decisions.	4.19 (0.617)	4.10 (0.658)	−0.10	−0.12
Q7. I could express my views and lead my own reasoning process.	4.50 (0.481)	4.38 (0.522)	−0.12	−0.17
Perceived support				
Q8. Virtual students prompted deeper thinking and participation.	4.38 (0.544)	4.33 (0.508)	−0.05	−0.07
Q9. The tutor supported me and kept the discussion on track.	4.31 (0.444)	4.19 (0.440)	−0.12	−0.18
Q10. The system provided timely prompts/corrections when needed.	4.35 (0.380)	4.29 (0.299)	−0.06	−0.10
Self-efficacy				
Q11. I felt more confident analyzing the case and justifying a diagnosis.	4.35 (0.457)	4.29 (0.395)	−0.06	−0.09
Q12. I can apply the learned reasoning strategies to similar cases.	4.50 (0.404)	4.52 (0.345)	+0.02	+0.04
Cognitive load				
Q13. Completing my tasks required substantial mental effort.	3.69 (1.444)	3.38 (1.283)	−0.31	−0.27
Q14. I often felt information overload during the discussion.	3.15 (2.053)	2.76 (1.705)	−0.39	−0.28
Q15. The system prompts or interface often distracted my attention.	2.54 (1.556)	2.00 (0.381)	−0.54	−0.53

a Full-sample results constitute the main descriptive questionnaire analysis. Filtered results comprise the nonprespecified sensitivity analysis and exclude 5 questionnaires with no ratings below the scale midpoint (all 15 items ≥3); mean differences and Cohen *d* are computed as filtered minus full sample.

b PBL: problem-based learning.

Among the 26 MAPLE-CR intervention sessions, transcript-based formative assessment was summarized as supplementary process-oriented evidence aligned with the scaffolding framework across the 5 PBL-relevant dimensions (Figure S1 in Multimedia Appendix 4). KA and CR had the highest mean scores (4.4 and 3.7) and relatively high minima (3.5 and 3.0), suggesting that most learners articulated coherent and largely correct diagnostic reasoning. By contrast, interaction-oriented dimensions showed greater variability: CC and SR had lower minima (both 1.5), and AP had the lowest central tendency (mean 2.1; minimum 0.5). Together, these findings suggest that even when learners reason accurately, they may not consistently externalize that reasoning through structured discussion, synthesis, and proactive engagement—core behaviors in PBL. Notably, the CC dimension reflects learner responsiveness within AI-mediated tutor/peer exchanges rather than real peer-to-peer teamwork.

In addition, we examined convergent validity by comparing the automated formative scores with independent expert ratings across 26 sessions (3 raters per session; 0‐5 scale; Table S1 in Multimedia Appendix 4). The expert panel consisted of 3 attending physicians involved in postgraduate medical education, each with at least 10 years of clinical teaching experience and prior experience in PBL facilitation or CR assessment. Expert raters assessed deidentified session transcripts independently and were blinded to each other’s ratings; automated formative scores were not used during expert rating. For the 4 evaluator-scored dimensions, the human experts used the same construct definitions, evidence rules, and 0 to 5 scoring anchors as the evaluator agent; the complete materials are provided in Multimedia Appendix 1. Interrater reliability of the mean expert ratings was moderate to good across dimensions (ICC[A,3]=0.729‐0.851): AP 0.851, CR 0.766, KA 0.729, CC 0.807, and SR 0.796. We quantified agreement using Spearman rank correlations (ρ) between automated scores and the mean expert rating per session, and absolute agreement using MAE. Agreement analyses showed strong rank-order consistency for AP (ρ=0.88, *P*<.001; MAE=0.40), SR (ρ=0.93, *P*<.001; MAE=0.50), and CR (ρ=0.78, *P*=.007; MAE=0.45). KA demonstrated moderately high agreement (ρ=0.81, *P*=.005; MAE=0.47). CC exhibited comparatively weaker, though still positive, agreement (ρ=0.71, *P*=.02) and the largest absolute deviation (MAE=0.68). Overall, these results provide preliminary evidence of convergence between the automated assessment and expert judgments in this small, single-study sample, particularly for participation, reflection, and reasoning. Because the CC reference standard showed good reliability (ICC[A,3]=0.807), the comparatively weaker automated-expert agreement for this dimension suggests that communication-related behaviors may benefit from further rubric calibration and model refinement.

Figure S2 in Multimedia Appendix 4 presents an exploratory Spearman rank correlation analysis across session-level evaluation measures within the intervention group. Pairwise complete observations were used: correlations involving questionnaire composites used 21 matched sessions, whereas correlations among test scores and formative metrics used 26 sessions. The 3 perceived cognitive-load-related items (Q13-Q15) were summed as an exploratory cognitive load (COG) composite, and the remaining questionnaire items (Q1-Q12) were summed as an overall experience (EXP) composite. Baseline performance was strongly negatively associated with score gain (pretest score vs score gain, ρ=−0.86), consistent primarily with ceiling effects and regression to the mean. Several transcript-based formative dimensions were positively interrelated, especially AP-SR (*ρ*=0.77), CR-SR (*ρ*=0.88), and CC-SR (*ρ*=0.81), indicating that interactional, reasoning, reflective, and regulatory features tended to co-occur within MAPLE-CR sessions. In contrast, the questionnaire composites showed weak associations with formative metrics and knowledge outcomes (eg, COG-KA, ρ=−0.07; EXP-posttest, ρ=0.19), suggesting that perceived cognitive load and overall experience should be interpreted as subjective learner-experience indicators rather than direct proxies for performance or process quality. Because no multiplicity adjustment was applied, these correlations should be interpreted descriptively rather than as confirmatory evidence.

### Voluntary Follow-Up: Descriptive Repeated-Use Patterns

**Figure 5.**
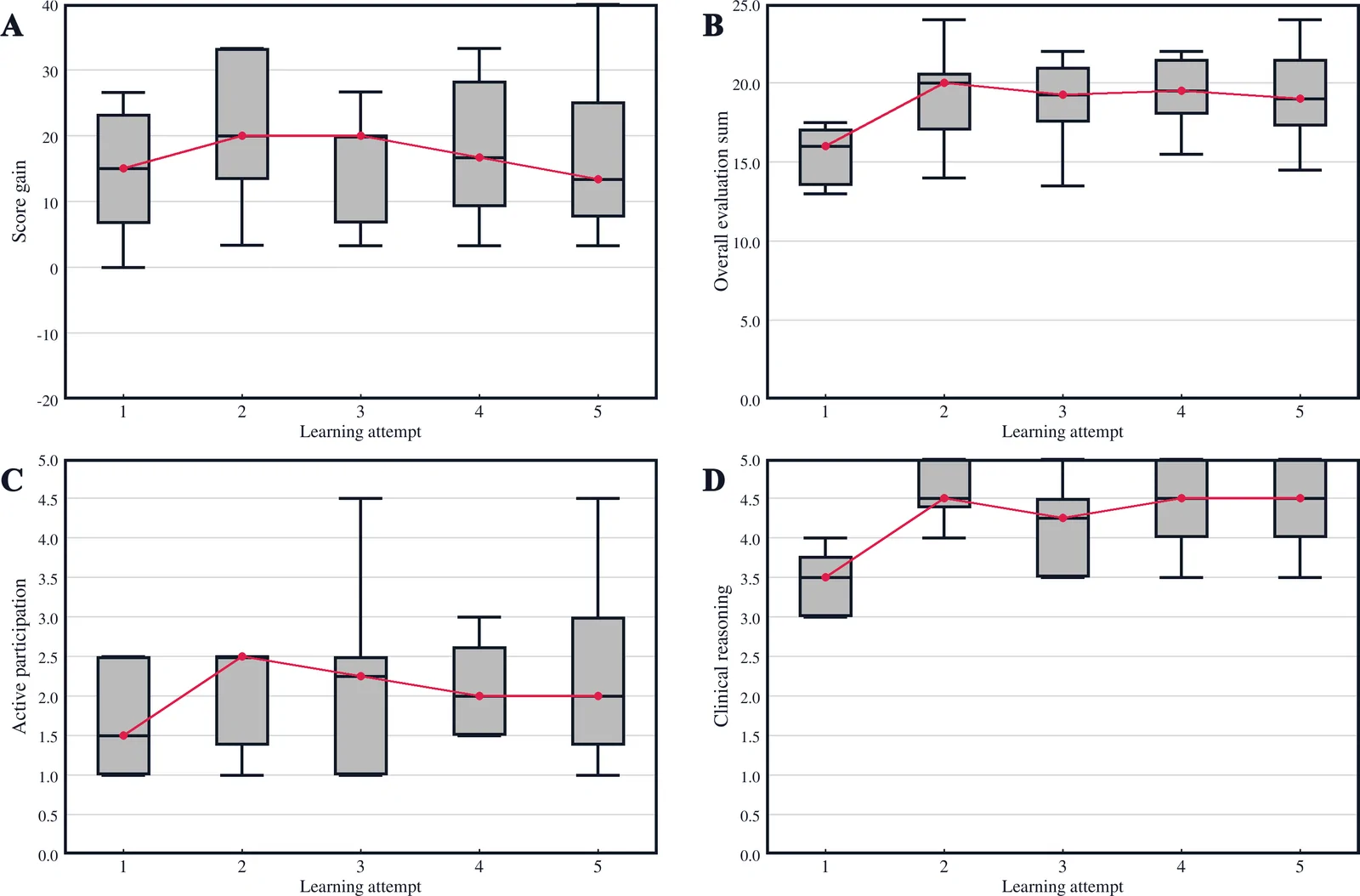
Descriptive repeated-use patterns across 5 Multiagent Problem-based Learning Environment for Clinical Reasoning (MAPLE-CR) attempts in the voluntary follow-up sample. Panels show (A) test score gain, (B) overall formative evaluation sum, (C) active participation, and (D) clinical reasoning. Box plots summarize the distribution at each attempt, with the center line indicating the median and the box spanning the 25th to 75th percentiles; the red line connects the median values across attempts to aid visual interpretation of the follow-up pattern.

We descriptively examined repeated-use patterns in within-session test score gain, CR, AP, and the overall evaluation sum across 5 attempts in the voluntary follow-up sample (Figure 5; n=18). Results for KA, CC, and SR are reported in Figure S3 in Multimedia Appendix 4.

Within-session pretest-to-posttest score differences were positive across all 5 attempts in the voluntary follow-up sample. The median within-session score differences were 15.00 (IQR 6.60-23.30; bootstrap 95% CI 8.30-21.65), 20.00 (IQR 13.30-33.30; bootstrap 95% CI 13.30-26.65), 20.00 (IQR 6.70-20.00; bootstrap 95% CI 6.90-20.00), 16.70 (IQR 9.18-28.35; bootstrap 95% CI 10.00-26.70), and 13.40 (IQR 7.60-25.20; bootstrap 95% CI 8.95-23.50) across attempts 1 through 5, respectively. All CIs were above zero, indicating positive within-session score differences at each attempt.

For formative outcomes, CR increased markedly from attempt 1 to attempt 2 (median 3.50 to 4.50; Hodges-Lehmann median difference=1.00, 95% CI 1.00‐1.50; Wilcoxon *P*<.001; Cohen *d*=1.739), indicating a higher transcript-based CR score at attempt 2 than at attempt 1 in this descriptive follow-up analysis. In contrast, AP showed a smaller, nonsignificant change (median 1.50 to 2.50; Hodges-Lehmann median difference=0.19, 95% CI 0.00‐1.00; Wilcoxon *P*=.15; *d*=0.411), indicating a smaller and more heterogeneous observed change in engagement. The overall evaluation sum also increased from attempt 1 to attempt 2 (median 16.00 to 20.00; Hodges-Lehmann median difference=3.50, 95% CI 2.50‐5.50; Wilcoxon *P*<.001; *d*=1.292), indicating a higher aggregate formative score at attempt 2 than at attempt 1.

From attempt 2 onward, no statistically significant within-subject trends were detected for CR (Friedman χ²_3_=6.484*, P*=.09), AP (Friedm*a*n χ²_3_=2.088, *P*=.55), or the overall *s*um (Friedman χ²_3_=3.034, *P*=.39). These nonsignificant results should not be interpreted as evidence of stabilization; rather, they indicate that no statistically significant later trend was detected in this small voluntary follow-up sample. A sample size sensitivity calculation indicated that detecting a smaller but educationally meaningful within-participant change of *d*=0.50 with 2-sided α=.05 and 80% power would require approximately 34 completed follow-up participants, exceeding the available follow-up sample (n=18).

Additionally, student case-study pathways are visualized in Figure S4 in Multimedia Appendix 4. Although all learners began with the same thoracic and airway surgery case, half diversified into other systems in the second attempt. By the fifth attempt, students had collectively explored over 9 distinct medical domains, with some choosing to revisit similar systems (eg, hepatobiliary or cardiovascular) and others branching into entirely new areas (eg, pediatrics or oncology). These patterns suggest heterogeneity in follow-up case selection; whether this reflected learner interests, perceived needs, or recommendation effects could not be determined from the present data.

We also recorded the time learners spent on each PBL discussion, excluding testing and assessment (Figure S5 in Multimedia Appendix 4). The overall median was 67.39 (IQR 54.7-90.0) minutes. The median discussion duration decreased from 70.87 (IQR 58.5-93.2) minutes at attempt 1 to 54.87 (IQR 48.1-82.9) minutes at attempt 5, a 22.6% reduction. Despite session-to-session fluctuations, discussions were shorter by the fifth attempt; however, this pattern may reflect increasing familiarity, case differences, or reduced engagement.

### Open-Ended Questionnaire

To contextualize learners’ perceived strengths and limitations of MAPLE-CR, we descriptively summarized the open-ended feedback entries and grouped them into nonmutually exclusive categories. The complete categorized comments are provided in Multimedia Appendix 5.

Positive feedback most often described structured CR support (5 feedback entries), perceived learning gains (4 entries), reduced pressure and freer expression compared with classroom PBL (4 entries), and content coverage or case realism (3 entries). These comments suggested that learners perceived the simulated PBL format as immersive, flexible, and helpful for organizing diagnostic and management reasoning.

Areas for improvement included interaction depth, flow, or tutor questioning (3 feedback entries), assessment or questionnaire design (2 entries), content repetition or concision (2 entries), and interface usability or navigation (2 entries). Single feedback entries also noted conversational accuracy or AI misunderstanding, time or session-control burden, and requests for additional learning-tool support such as discussion filtering or mind-map functions.

Overall, these open-ended responses provided descriptive support for perceived usefulness and learner acceptability while identifying concrete areas for refinement in conversational accuracy, interaction design, assessment design, and workload.

## Discussion

### Principal Findings

This exploratory randomized educational evaluation provides preliminary evidence that MAPLE-CR is a feasible and educationally valuable AI-supported PBL environment for CR training. In the randomized component, learners using MAPLE-CR achieved higher posttest scores and greater pretest-posttest gains than those in the self-study control, suggesting a large short-term difference under the present study conditions rather than definitive efficacy evidence. Learner-reported outcomes indicated high acceptability and perceived educational value across satisfaction, involvement, perceived support, and self-efficacy. Because engagement with generative AI may vary with learner characteristics and implementation context, these acceptability findings should be interpreted within the present population and setting [30,31]. Notably, however, learners also reported relatively high ratings on cognitive-load items. This pattern should not be interpreted as evidence that reducing real peer coordination is educationally beneficial or that AI-mediated PBL can replace human group interaction. Rather, for MAPLE-CR as a supplementary PBL learning environment, the design challenge is to preserve productive reasoning and dialogic engagement while minimizing avoidable interface or workflow demands. From a cognitive load perspective, this means preserving productive challenge associated with reasoning and explanation [32,33] while reducing extraneous load introduced by the learning interface and workflow structure [34]. From a social constructivist perspective, these findings are consistent with the possibility that AI-driven scaffolding can function as a provisional bridge within the learner’s Zone of Proximal Development [35]. One interpretation is that MAPLE-CR’s orchestration of the PBL process, including structured summaries, next-step prompts, and feedback loops, may have helped learners direct effort toward higher-level reasoning, although the specific contribution of each scaffolding component requires further investigation.

The control condition produced a measurable median gain of +6.70 (IQR 0.00-13.40) points, whereas the between-group median difference in gain was 6.60 points (bootstrap 95% CI 1.60-14.95). Using these values as a descriptive anchor, a substantial portion of the short-term pretest-posttest improvement was also observed under structured exposure to the same case materials, while the additional separation represents the incremental difference observed for MAPLE-CR under this comparator. This comparison is descriptive only and should not be interpreted as a causal partitioning of the effect. The present design evaluated MAPLE-CR as an integrated learning package. The observed between-group difference may therefore reflect the combined influence of platform use, orientation, AI-mediated interaction and feedback, additional structured exposure, cognitive and regulatory scaffolding, novelty, and motivational engagement rather than the isolated effect of the AI-PBL scaffolding mechanisms; nor does the design establish noninferiority to faculty-facilitated, peer-driven PBL.

Across the voluntary 4-week follow-up, descriptive patterns showed higher CR and aggregate formative scores at attempt 2 than at attempt 1, followed by statistically uncertain later trajectories. Given the absence of a control group, self-selected participation, heterogeneous case pathways, and the small sample, these observations do not establish maintenance, stabilization, or sustained learning; they could reflect adaptation, ceiling or novelty effects, effort, case heterogeneity, or limited power [36,37]. They instead support the feasibility of repeated MAPLE-CR use across different cases and identify patterns that warrant evaluation in controlled longitudinal studies, including studies of adaptive prompts or progressively more complex cases [21].

In addition, a key pattern was the dissociation between relatively strong knowledge/reasoning performance and more heterogeneous interactional performance (eg, participation, collaboration, and reflection). This aligns with prior work suggesting that competence in CR does not automatically translate into observable group-process skills, particularly in technology-mediated settings where turn-taking norms and role clarity are less salient than in facilitator-led discussions [22,38]. Accordingly, AI-enabled PBL designs may need to scaffold not only what learners think (eg, prompts for hypothesis generation and justification) but also how learners participate and co-construct understanding (eg, turn-taking, accountability structures, and synthesis routines). Within the present study, MAPLE-CR represents an empirically evaluated implementation of conversational AI that spans multiple phases of the PBL cycle rather than serving as an occasional question-answering aid. The findings also indicate where additional process support is needed.

### Implications for Medical Education

Beyond our local internship context, a key design implication of MAPLE-CR is a set of potentially transferable scaffolding features for supporting scalable CR practice: embedding cognitive prompts that require explanation and revision, social structures that elicit multivoice challenge rather than simple learner-AI dyads, and regulatory supports that make phases and feedback loops explicit. When trained facilitators, standardized patients, or stable small-group scheduling are difficult to sustain, such a pattern may offer a scalable supplement for presession rehearsal, between-session deliberate practice, or postsession consolidation within blended curricula [39]. Because simulated group-process behaviors varied across sessions, AI is unlikely to fully reproduce the nuance of human facilitation [23]; implementation may therefore benefit from brief learner orientation (expectations for participation, justification, and turn-taking) and a short postsession debrief to normalize reflection and calibrate engagement. In future implementations, transcript summaries and formative analytics could be evaluated as tools to help faculty identify sessions with recurrent needs (eg, persistently low collaboration or superficial justification) and provide focused coaching without routine full-transcript review. At its current maturity, MAPLE-CR is best positioned as a scalable, formative, low-stakes supplement for structured PBL-like practice rather than as a replacement for faculty-facilitated PBL; further multisite evidence is needed before considering high-stakes or summative uses, particularly across languages, curricula, and assessment regimes.

### Safety

MAPLE-CR is an educational tool rather than a clinical decision support system and is intended for formative, low-stakes learning rather than summative decision-making. Tutor and evaluator functions are bounded by case objectives and curated learning resources, and feedback emphasizes reasoning processes (eg, justification, revision, reflection) rather than prescriptive management recommendations. Cases and prompts are designed for training purposes and do not involve identifiable patient information; discussion transcripts and learning records are stored and analyzed in deidentified form for educational feedback and research. Although no safety incidents were observed in this study, future iterations will strengthen safeguards to mitigate misinformation and over-reliance and will maintain ongoing monitoring and a clear reporting pathway for any concerning outputs [10,40,41].

### Limitations and Suggestions for Future Research

This study has limitations. First, no formal a priori sample size calculation was conducted. Although the sensitivity analysis showed that the observed effect magnitude exceeded the approximate large-effect threshold detectable with the available sample, this descriptive analysis does not establish prospective power sufficiency; the study was not prospectively powered as a confirmatory efficacy trial, and the precision of the estimated effect remains limited. The findings should therefore be confirmed in larger, prospectively powered, multicenter trials with stronger active comparators. The adapted postsession questionnaire was not validated specifically for AI-supported PBL, so learner-experience findings should be interpreted descriptively. Second, the trial was retrospectively registered, which limits full compliance with prospective RCT registration expectations and should be considered when interpreting the randomized component. Third, the control condition was self-study, which represents a relatively modest active comparator; this comparator did not isolate the contribution of specific PBL scaffolding mechanisms from other features of MAPLE-CR, such as AI-mediated dialogue, simulated peer/tutor interaction, novelty, or motivational engagement; future studies should benchmark MAPLE-CR against stronger comparators that more closely reflect established PBL pedagogy (eg, facilitator-led small-group PBL), near-peer facilitation, or other structured instructional modalities [15,42]. The conditions were also not matched for total structured exposure because only the MAPLE-CR group received the 40-minute orientation. The randomized component also used a single standardized lung cancer/thoracic-airway surgery case to ensure comparable exposure across groups; therefore, the observed effect may not generalize across clinical domains, case complexity levels, or scenario types. The voluntary follow-up supported repeated use across additional cases but was not designed to determine whether MAPLE-CR effects differ by case type or complexity. In addition, stochastic LLM generation may have introduced variability in the tutor and peer responses that learners received, creating a potential source of within-condition interference that was not explicitly modeled. Fourth, outcomes emphasized immediate knowledge performance and learner experience; although the follow-up suggested feasibility for repeated cross-case use, participation was voluntary and may overestimate engagement and perceived benefit. The follow-up included only 18 participants and was not powered for confirmatory inference about longer-term learning trajectories. Fifth, we did not include standardized external measures of retention and transfer (eg, objective structured clinical examination-style assessments) [43]. Although the transcript-derived formative dimensions were aligned with the proposed scaffolding framework and showed preliminary convergence with expert ratings, they were not prospectively designed to test mediation or isolate specific cognitive, social-interactional, or regulatory mechanisms. Future studies should incorporate prespecified process measures to examine how specific scaffolding functions contribute to learning outcomes.

Beyond these study-level limitations, future research should move from asking whether AI-supported PBL “works” to testing how and for whom it works. Mechanism-focused studies could examine whether improvements are mediated by process indicators aligned to the proposed cognitive, social-interactional, and regulatory functions (eg, quality of justification and revision, participation patterns and responsiveness, adherence to PBL phases, and uptake of feedback across cases). Work is also needed to identify boundary conditions and learner-level moderators (eg, baseline knowledge, self-efficacy, language/communication confidence, and AI literacy), given the observed heterogeneity in group-process behaviors. Finally, the higher scores observed at attempt 2 and statistically uncertain later trajectories suggest a need to investigate adaptive progression and scaffold fading—such as progressively more complex cases, calibrated prompts, or feedback designs—under controlled longitudinal conditions [24]. Multisite and real-world implementation studies should evaluate feasibility, faculty workload, and sustainability alongside learning outcomes, and clarify appropriate use as formative support rather than high-stakes assessment.

### Conclusions

This study provides preliminary evidence that MAPLE-CR is a viable AI-supported scaffold for PBL and suggests that generative AI can serve a pedagogical role beyond functioning as a mere information source. MAPLE-CR was associated with larger short-term knowledge gains than self-study and showed high acceptability and feasible repeated use, but the design did not isolate specific scaffolding mechanisms. At the same time, the finding that gains in knowledge and CR may emerge earlier than improvements in observable participation and collaborative process skills highlights the complexity of AI-mediated instructional design. As a scalable supplement, MAPLE-CR may provide additional opportunities for structured PBL-like practice when faculty facilitation and repeated small-group sessions are difficult to sustain. These findings require confirmation in larger, prospectively powered, multicenter trials with stronger active comparators and longer-term measures of retention, transfer, and cross-case generalizability.

## Supplementary material

10.2196/95039 Multimedia Appendix 1 Detailed description of the technical implementation of multiagent problem-based learning environment for clinical reasoning.

10.2196/95039 Multimedia Appendix 2 Video demonstration of the MAPLE-CR system workflow and user interface.

10.2196/95039 Multimedia Appendix 3 Blueprint of the pretest and posttest design.

10.2196/95039 Multimedia Appendix 4 Additional experimental and follow-up results.

10.2196/95039 Multimedia Appendix 5 Descriptive categorization of open-ended feedback.

10.2196/95039 Checklist 1 CONSORT-eHEALTH checklist (V 1.6.1).

## Abbreviations

AP: active participation
CC: communication and collaboration
CONSORT-eHEALTH: Consolidated Standards of Reporting Trials of Electronic and Mobile Health Applications and Online Telehealth
CR: clinical reasoning
ICC: intraclass correlation coefficient
KA: knowledge accuracy
LLM: large language model
MAE: mean absolute error
MAPLE-CR: Multiagent Problem-based Learning Environment for Clinical Reasoning
PBL: problem-based learning
SR: summarization and reflection
