# Supplementary material for: Scaffolded AI-Supported Problem-Based Learning for Medical Interns: Exploratory Retrospectively Registered Randomized Controlled Evaluation With a Voluntary Feasibility Follow-Up
Source: JMIR Med Educ. 2026 Sep 3;12:e95039. doi: 10.2196/95039 (PMC13541230; doi:10.2196/95039)
Supplement: Supplementary file 1 [file mededu-v12-e95039-s004.pdf]

Additional experimental and follow-up results.

1 Additional results

Dimension	Expert Mean (SD)	Spearman ρ	95% CI	p-value	MAE
Active Participation (AP)	2.25 (1.27)	0.88	[0.54, 0.99]	<.001	0.40
Clinical Reasoning (CR)	3.36 (0.39)	0.78	[0.39, 0.95]	.007	0.45
Knowledge Accuracy (KA)	3.61 (0.74)	0.81	[0.33, 0.98]	.005	0.47
Communication and Collaboration (CC)	2.96 (0.33)	0.71	[0.15, 0.93]	.022	0.68
Summarization and Reflection (SR)	3.10 (0.51)	0.93	[0.71, 1.00]	<.001	0.50

Table S1. Convergent validity of automated assessment vs expert ratings across 26 rated sessions (3 experts/session; 0–5 scale). Expert scores averaged across raters before agreement analyses.

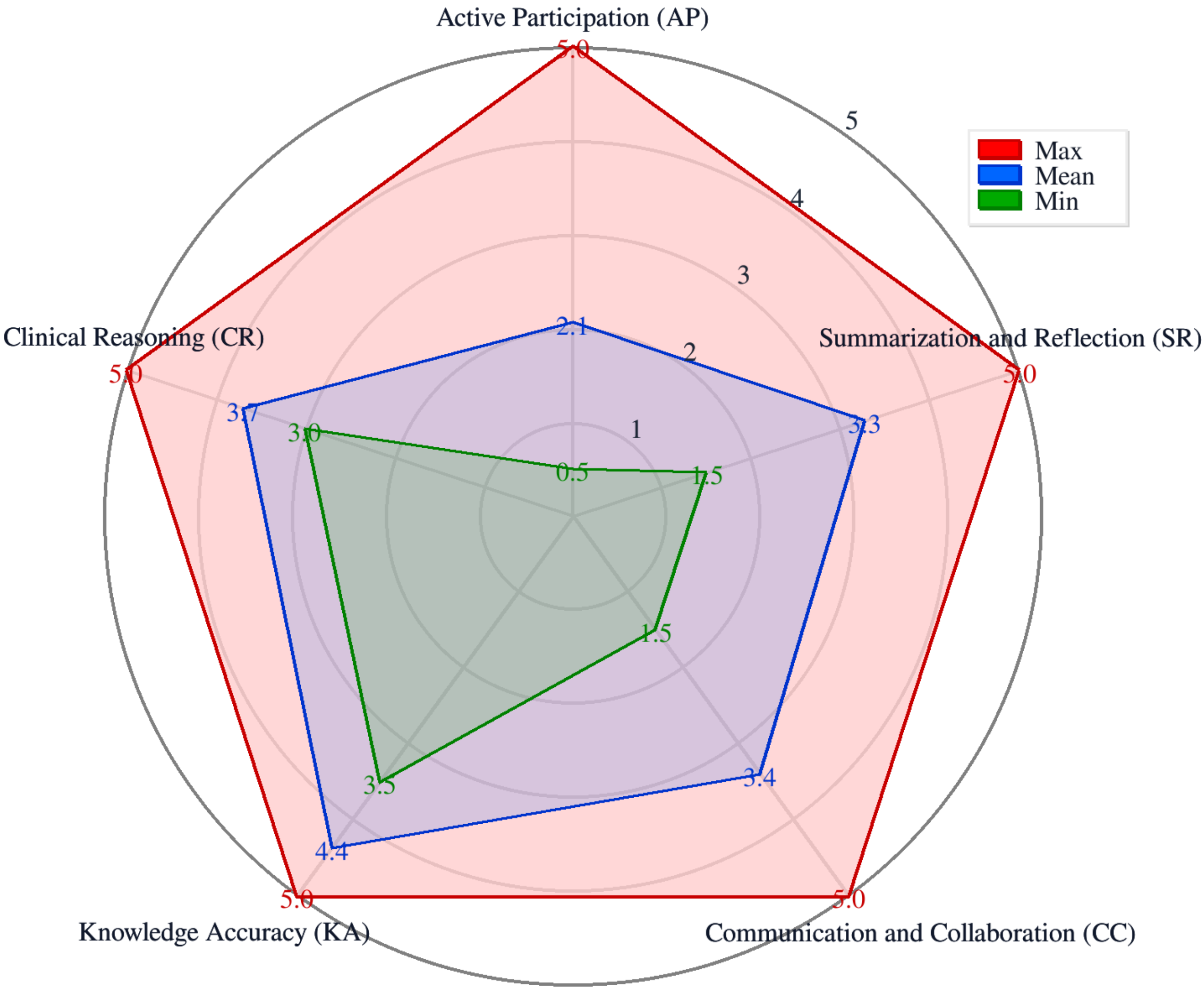

Figure S1. Radar summary of automated formative assessment across five dimensions. Scores were rated from 0 to 5 in 0.5-point increments; the plot shows participant-level minima/maxima and the mean for each dimension.

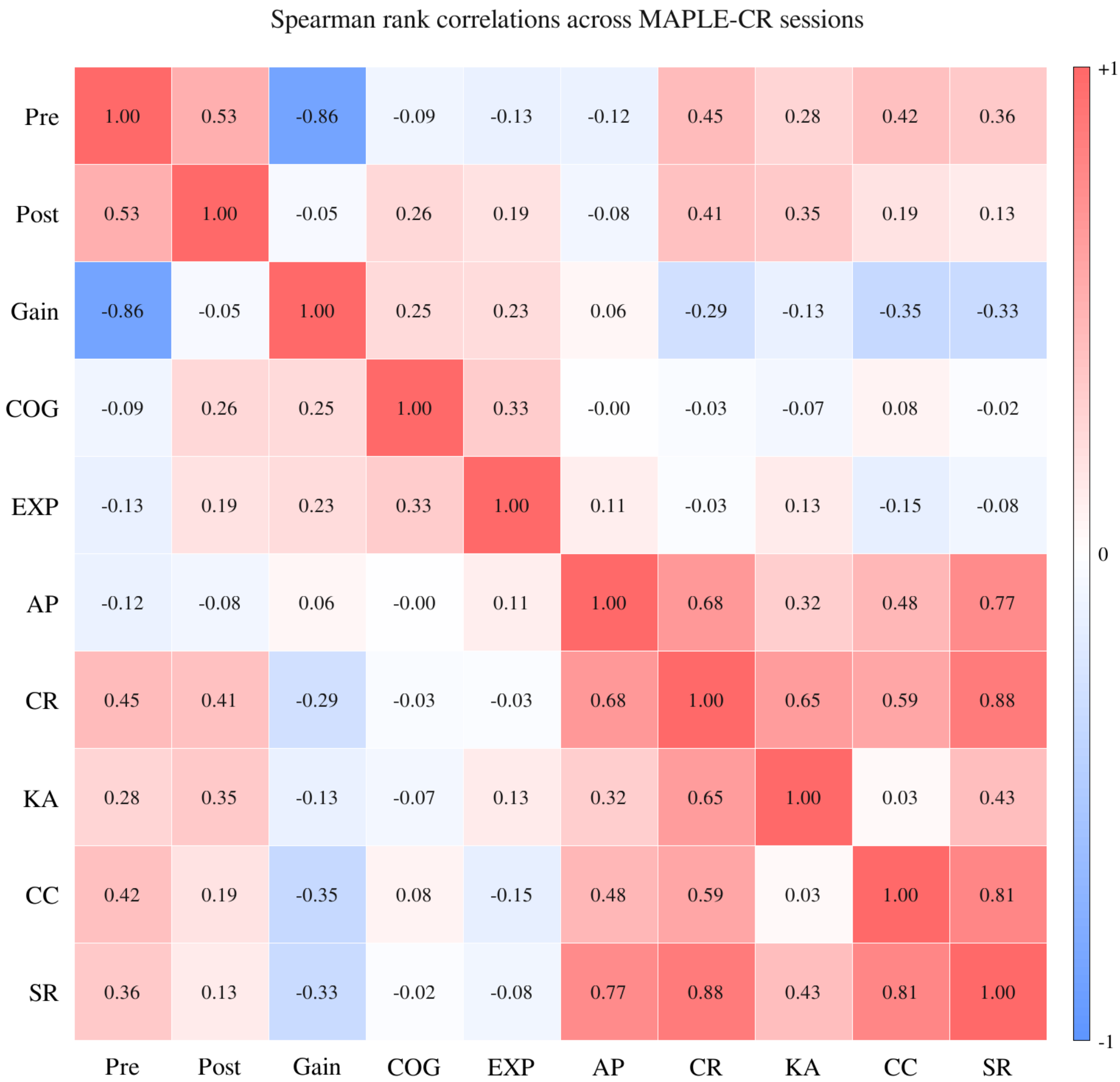

Figure S2. Correlation heatmap of summative outcomes, formative metrics, and questionnaire composites (COG: sum of cognitive-load items; EXP: sum of non-cognitive-load items).

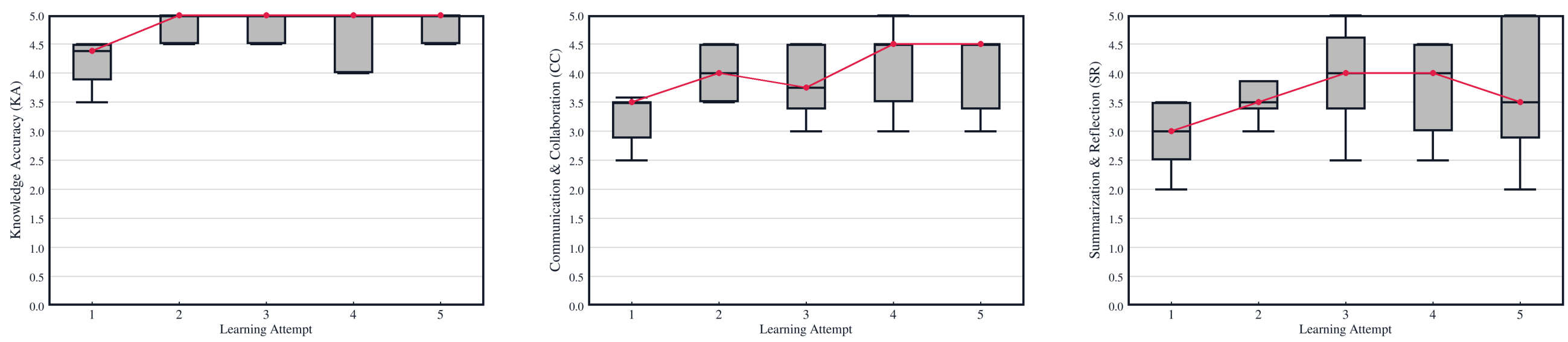

(a) (b) (c)

Figure S3. Descriptive patterns in KA, CC, and SR across five MAPLE-CR attempts.

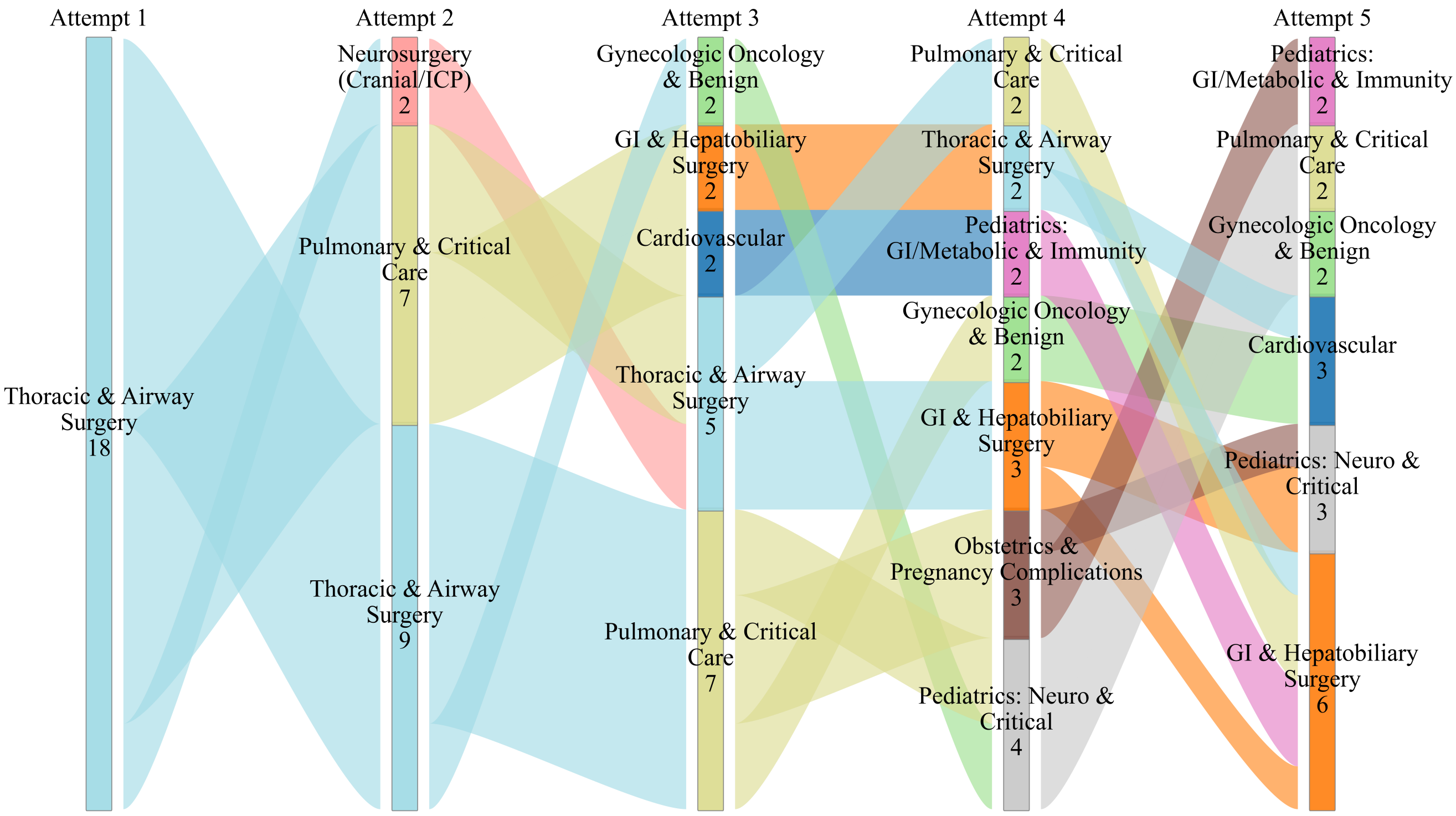

Figure S4. Learners' case-selection pathways across five MAPLE-CR sessions.

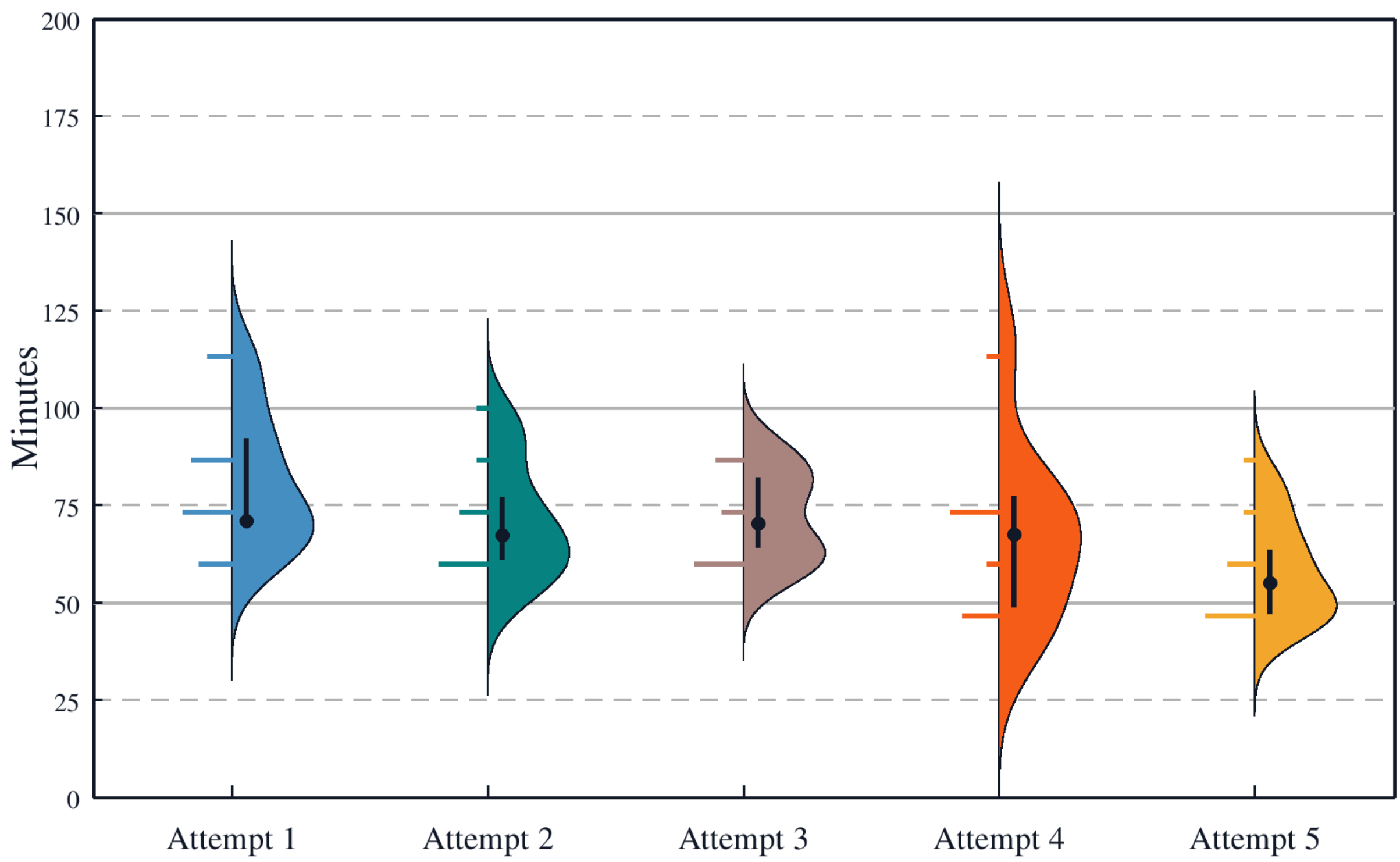

Figure S5. Distribution of Study times by attempts.
