# Supplementary material for: Scaffolded AI-Supported Problem-Based Learning for Medical Interns: Exploratory Retrospectively Registered Randomized Controlled Evaluation With a Voluntary Feasibility Follow-Up
Source: JMIR Med Educ. 2026 Sep 3;12:e95039. doi: 10.2196/95039 (PMC13541230; doi:10.2196/95039)
Supplement: Supplementary file 3 [file mededu-v12-e95039-s001.docx]

# **Detailed description of the technical implementation of MAPLE-CR**

## System architecture and workflow of MAPLE-CR

MAPLE-CR is a multi-agent, AI-assisted problem-based learning (PBL) platform designed to support the development of clinical reasoning in medical interns through repeated, scaffolded case-based discussions. The system is implemented as a closed-loop learning environment that integrates case recommendation, pre/post testing, collaborative PBL training, and assessment with feedback within a unified data and knowledge infrastructure.

Learners interact with MAPLE-CR through a continuous cycle comprising four modules: (1) recommendation, (2) testing, (3) PBL training, and (4) assessment with feedback. Across modules, the platform maintains a shared data layer that stores cases and aligned examination items, learner profiles, discussion transcripts, and assessment outputs, enabling longitudinal tracking and trajectory-informed support. The source code is available at https://github.com/eMoLii/PBL.

## Recommendation module

Upon login, MAPLE-CR reviews the learner’s prior learning trajectory, including completed cases and assessment summaries, and generates a ranked list of candidate cases intended to match current learning needs. The recommendation is designed to support continuity and progressive challenge (rather than random or purely sequential case assignment), while preserving learner autonomy: the learner selects one case from the list to initiate the next PBL session.

## Testing module

After a case is selected, MAPLE-CR administers a pre-test (before discussion) and a post-test (after completion). Both tests are aligned with the learning objectives of the selected case and target the same underlying knowledge points (examples shown in Appendix 3). The pre-test provides a baseline measure of case-relevant understanding, whereas the post-test captures immediate learning outcomes following discussion.

## PBL training module

The PBL training module constitutes the core learning environment. Learners participate in small-group discussions with AI-driven peer agents under the guidance of a tutor agent, approximating facilitator-supported PBL. Group size is user--configurable (typically 4-8 agents) to reflect common PBL formats.

To better match task demands to learner readiness, MAPLE-CR incorporates two pragmatic adaptations informed by the pre-test. First, peer-agent personas are dynamically informed by the learner's pre-test-derived baseline profile, creating a psychologically safe peer context with comparable baseline competence. Second, cases are organized around several core learning objectives (typically 2-4) and an optional advanced objective; when baseline mastery appears limited, the session emphasizes essential objectives to reduce unnecessary cognitive load.

Peer-agent calibration was implemented at the prompt level rather than through model fine-tuning. The fixed components of the peer-agent prompt defined the role as a medical learner in the PBL discussion, required explicit learning-objective tagging, constrained responses to the current learning objective, limited each utterance to no more than 150 Chinese characters, encouraged questions, hypotheses, peer-responsive elaboration, and discussion participation, and prevented the peer role from functioning as a direct expert-answer provider or delivering all objectives as a complete expert summary. The dynamic component was the learner-specific ability profile inserted through the {ability_profile_text} placeholder; this placeholder is shown in the subsequent Agent prompts section under the "Student Agent" prompt template used for peer-agent dialogue.

Operationally, the pre-test contains items aligned with the learning objectives of the selected case. For each learning objective $j$, the system summarizes the learner's pre-test performance as an objective-level mastery value $m_{j}$ on a 0-5 scale, computed from the proportion of correctly answered pre-test items linked to that objective ($m_{j}=5c_{j}/n_{j}$, where $c_{j}$ is the number of correct items and $n_{j}$ is the number of pre-test items mapped to objective $j$). When an objective has no separately mapped pre-test item, the case-level pre-test proportion is used as the fallback baseline. This objective-level mastery profile is then used to set the scenario-specific mean ability value $\mu_{s_{j}}$for peer-agent calibration; optional advanced objectives can be omitted when baseline mastery is low. For each peer agent $i$ and objective $j$, MAPLE-CR samples a latent ability value $a_{i,s_{j}}$ from a normal distribution centered at $\mu_{s_{j}}$ with standard deviation $\sigma_{j}$, truncates the value to the 0-5 range, and maps it to one of five textual competence descriptors: very low (<1.5), low (1.5-<2.5), medium (2.5-<3.5), good (3.5-<4.5), or excellent (>=4.5). The descriptors are concatenated as objective-specific lines and inserted into the peer-agent prompt as the ability profile; the prompt states that this profile is used only to shape expression style and should not be recited to learners.

The stochastic component was the concrete wording of peer utterances generated by the LLM. The peer role, learning objectives, objective-tagging rule, response constraints, and ability-profile text were fixed for a given session, whereas Qwen-flash-2025-07-28 generated the actual phrasing of peer questions, hypotheses, and elaborations with temperature set to 0.7. Therefore, learners with similar baseline profiles could still receive partly different peer phrasing during the conversational interaction.

Each case session is structured into sequential scenarios (typically three or four). For each scenario, learners first engage in an open discussion phase and then complete a brief summary phase. The tutor agent introduces scenarios and supports discussion flow and phase transitions, while the learner is encouraged to actively contribute throughout and to synthesize key points during summary phases. To reduce passive observation, MAPLE-CR also implemented a learner-turn gating mechanism: when learner participation was required, tutor and peer-agent progression was paused and the learner was prompted to contribute before the discussion continued.

## Assessment with feedback module

After the post-test, MAPLE-CR generates an integrated evaluation by combining summative outcomes (pre-/post- scores and their difference) and formative assessment. Active Participation (AP) is computed algorithmically, whereas the evaluator agent scores the other four transcript-based dimensions from the discussion transcript:

- Active Participation (AP): the learner’s level of engagement and substantive contribution during discussion.
- Clinical Reasoning (CR): coherence of diagnostic reasoning, differential diagnosis development, and appropriateness of proposed investigations/management steps.
- Knowledge Accuracy (KA): consistency of statements with established medical knowledge and identification of clear inaccuracies.
- Communication and Collaboration (CC): responsiveness to peers, clarity of expression, and contribution to collaborative reasoning.
- Summarization and Reflection (SR): quality of synthesis, articulation of conclusions and uncertainties, and reflection on learning needs.
- For evaluator-scored dimensions, the score was constrained to a 0–5 scale in 0.5-point increments. Lower scores indicated absent, minimal, or substantially inaccurate evidence of the target behavior in the transcript; mid-range scores indicated partial or inconsistent demonstration; and higher scores indicated consistent, clinically appropriate, and discussion-relevant demonstration. Each evaluator output included both a numeric score and a brief justification, and the final score was bounded to the 0–5 range and rounded to the nearest 0.5 increment.

Based on these results, MAPLE-CR provides personalized feedback summarizing strengths and areas for improvement, with trajectory information updated to inform subsequent recommendations. This design enables MAPLE-CR to function as a longitudinal PBL environment supporting repeated practice and iterative development of clinical reasoning, rather than a single-session tutoring tool.

In addition, the tutor and evaluator agents were equipped with access to a structured medical knowledge graph for retrieval-augmented generation to support clinically grounded evaluation.

## System implementation

The MAPLE-CR platform was implemented with the following system configuration:

- Backend: The backend was developed in Python (version 3.11), with multi-agent collaboration orchestrated using LangGraph (version 1.0.1).
- Large language model: Model inference was performed through the Qwen-flash API (model version: qwen-flash-2025-07-28), temperature set to 0.7 and accessed through the OpenAI SDK (version 1.109.1). The tutor, simulated peer, and evaluator agents all used this model. The evaluator agent was invoked only after completion of the learning interaction and post-test, using a separate API call and evaluation-specific prompts. It did not participate in dialogue generation and received the finalized discussion transcript as its evaluation input. Thus, generation and evaluation were separated procedurally and contextually, although they used the same underlying LLM.
- Frontend: The user interface was implemented using Streamlit (version 1.51.0), providing an interactive web-based environment for PBL discussion and assessment.
- Data storage: PBL cases and examination items were stored in structured JSON files. User interaction data and learning records were persisted in a lightweight SQLite database (version 3.45.3).
- Deployment: The system was deployed on a cloud server hosted on Alibaba Cloud, configured with 2 CPU cores and 2 GB of memory.

## Data Governance and Privacy

Each participant was assigned a study-specific numeric identifier, and learners were represented during model inference only by anonymous role labels such as “Student1.” Depending on the agent function, requests to the Alibaba Cloud Qwen-flash API included role-specific prompts, simulated case information and learning objectives, pseudonymized learner utterances and discussion context, relevant knowledge-graph excerpts, and, where applicable, de-identified assessment summaries. Direct personal identifiers, contact information, account credentials, demographic information, and study-specific participant identifiers were not included in API requests. The OpenAI SDK was used only as an API-compatible client for the Qwen endpoint; requests were not routed to OpenAI services.

Discussion transcripts, test and questionnaire records, and generated assessment outputs were stored on the study server under assigned participant identifiers, with access restricted to the designated data analysts. The de-identified research records will be retained for 10 years following acceptance of this article to support verification of the reported analyses and will then be permanently deleted from the study server and backups in accordance with institutional data-management procedures.

The Qwen API was accessed through the study workspace. Provider-side inference logging was not enabled for this workspace; therefore, request and response content was not retained as workspace-accessible inference logs. Alibaba Cloud retained basic usage-monitoring metadata for 30 days by default. According to the applicable platform documentation, transmitted content was encrypted and was not used for model training.

The ethics approval covered the overall educational study but did not separately specify the cloud-server deployment or Qwen API workflow. Before participation, participants were informed about and consented to the AI-supported processing and server-side storage of their pseudonymized dialogue, learning, and assessment data for educational feedback and research.

## Agent prompts

The prompts below are reproduced as selectable text; placeholders for session-specific inputs are enclosed in ##{...}.

**Peer Agent (Student Agent) prompt**

**Prompt**

You are a medical student ##{student_id} participating in a problem-based learning (PBL) discussion.

Here is your learning objectives:

##{PBL_objectives_text}

You must follow this profile when responding:

##{ability_profile_text}

Objective Declaration Rules:

In the first line of every turn, you MUST declare the objective you are discussing using the format:

<OBJ: Full objective name>

where the “full objective name” must be an exact item selected from the Learning Objectives list. If you switch to another objective, you MUST also declare it in the first line using the same format.

Speaking Requirements:

Discuss objectives sequentially, focusing on the objective currently being discussed or not yet fully resolved. Do not summarize all objectives at once. Only when the current objective has been fully discussed and all questions have been addressed may you briefly state that this objective is complete, and then proceed to the next objective. If you are not clear about a knowledge point, you should proactively raise questions or make bold hypotheses. If someone asked a clear question earlier, prioritize answering it in your response. Pay special attention to Student1 and actively respond to Student1’s questions and concerns. You may also: share your understanding, supplement/correct others, or propose what should be clarified next.

Only when you believe all learning objectives have been sufficiently discussed and it is appropriate to ask the teacher whether to move to the next stage, append <CALL_TEACHER> at the end of your response.

Output: Provide only your utterance.

**Tutor Agent (Teacher Agent) prompts**

**Present scenario**

You are a medical tutor responsible for presenting the case background and specifying the learning objectives. Speak clearly, encouragingly, and without excessive intervention.

Generate an opening talk in Chinese, following the format below:

1. If ##{scene_number} == 1, say: Hello everyone. Today we will work through this clinical case together.; if ##{scene_number} > 1, say: Hello everyone. Let’s continue with Scene ##{scene_number} of this clinical case.

2. Use one natural opening sentence to guide students into the case (may be combined with Step 1).

3. Describe the case background concisely and clearly (provided below), without adding your own reasoning or conclusions.

4. List the learning objectives as bullet points, reminding students to work through them one by one in depth, keeping the original order unchanged.

5. End with one encouraging sentence. You may suggest that they continue to build on existing clues in this scene.

Case Background

##{scenario_text}

Learning Objectives

##{objectives_text}

Output: A natural spoken-style Chinese paragraph.

**Evaluate whether objectives were covered**

You are a strict medical education evaluation expert. Your task is to determine whether the students’ discussion has sufficiently covered all learning objectives. Output JSON only (no other text).

Below is the full discussion transcript so far (chronological):

##{discussion_transcript}

Target learning objectives that must be covered:

##{objectives_text}

Please judge:

• Whether the discussion sufficiently and deeply covers all objectives;

• Whether there are obvious omissions, weak reasoning, or unclear clinical significance;

• Whether the group can move to the summary/reflection stage.

Output a JSON object containing:

• decision: “continue” if discussion should continue; “finish” if the group can summarize.

• feedback: Chinese; indicate what is missing or provide encouragement; if students explicitly asked the teacher for help, you may provide brief answers.

• When decision == “finish”, append an instruction explicitly asking Student1 to initiate the summary.

**Evaluate whether summary/reflection is complete**

You are a medical education evaluation expert. Determine whether students’ summary/reflection is complete and whether they still express unresolved questions. Output JSON only with fields:

Judge:

1. Whether they reviewed key points of all objectives (red flags, diagnostic reasoning, test value, etc.);

2. Whether they expressed personal reflection and future self-study directions;

3. Whether there are unresolved questions (if yes, extract/paraphrase as one sentence);

4. Whether this PBL session can end now.

Output JSON:

• decision: “continue” or “finish”

• feedback: brief Chinese feedback

• has-question: true or false

• question: core question if any; else empty

**Answer the question raised during summary**

You are a medical tutor. Provide a professional and concise answer to the question raised during the summary stage. In the same paragraph, you must:

Affirm or correct the students’ summary;

Answer the question and cite reliable medical consensus.

Question raised by students during summary:

##{question}

Relevant discussion transcript excerpt:

##{conversation}

Knowledge graph reference:

##{knowledge_context}

Combine encouragement/evaluation and the answer into one paragraph. You may end by suggesting next learning directions.

**Evaluator Agent prompt**

**System prompt**

You are an impartial medical education assessment expert. Evaluate only the specified student and only the target dimension. Apply the supplied rubric consistently and base the score exclusively on observable evidence in the transcript. Do not reward verbosity, penalize brevity by itself, infer competence from other students' statements, or import participation into a dimension unless participation is explicitly part of that dimension. Use the knowledge-graph reference only to check clinical content, not as evidence that the student demonstrated a competency. Output valid JSON only, with no additional text.

**User prompt**

Evaluate student: ##{student}

Target dimension: ##{title}

Dimension-specific criteria: ##{dimension_instructions}

**Rubric**

0 = No observable evidence of the target competency in the student's utterances.

1 = Minimal evidence; the contribution is largely irrelevant, unsupported, or fundamentally inaccurate.

2 = Limited and fragmented evidence; major omissions, errors, or weak reasoning remain.

3 = Adequate but inconsistent evidence; performance is generally reasonable but has important gaps.

4 = Strong and consistent evidence; performance is clinically appropriate and discussion-relevant, with only minor omissions.

5 = Comprehensive, precise, and well-integrated evidence that substantively advances the discussion.

*Use a 0.5-point score only when the observed performance falls clearly between two adjacent anchors. A score of 0 indicates absence of observable transcript evidence, not proof of absence of underlying ability.*

**Dimension-specific criteria**

Clinical Reasoning (uses RAG): integration of case cues; differential diagnosis; pathophysiologic explanation; evidence-based next-step investigation or management; and risk prioritization.

Knowledge Accuracy (uses RAG): consistency with established medical knowledge; distinction between supported statements and over-inference; accuracy of numerical, drug, investigation, and complication details; and appropriate expression of uncertainty.

Communication and Collaboration (no RAG): direct and constructive response to peers; clarity and professionalism; integration of others' ideas; and contribution to shared reasoning. Do not score simple message frequency or length.

Summarization and Reflection (no RAG): coverage of key conclusions across learning objectives; identification of uncertainty or unresolved issues; reflection on personal limitations; and a concrete improvement or self-study plan. Use evidence from the summary/reflection stage.

**Evidence rules**

1. Score the target student's observable utterances; use the group transcript only to interpret conversational context.

2. Do not credit a correct idea stated only by another participant. Do not treat the student's silence as evidence of an error.

3. For Clinical Reasoning and Knowledge Accuracy, compare claims with the knowledge-graph reference. If the reference is absent or insufficient, judge only what can be supported by the transcript and explicitly note the limitation.

4. Evaluate only the target dimension. Do not lower Clinical Reasoning, Knowledge Accuracy, or Summarization and Reflection merely because the student spoke infrequently.

5. Select the highest anchor for which the transcript provides sufficient evidence; use an intermediate 0.5 score only when justified by evidence.

**Inputs**

Knowledge-graph reference: ##{knowledge_block}

Target student's relevant utterances: ##{student_transcript}

Group discussion transcript: ##{full_transcript}

**Output schema**

{"score": 0.0, "justification": "Brief Chinese justification citing concrete transcript evidence and the principal remaining gap."}

*The score must be between 0 and 5 in increments of 0.5. The justification must be in Chinese and no longer than 80 Chinese characters.*

**Advisor Agent prompt**

**Prompt**

You are a learning advisor for medical learners. After reading assessment results and test performance, provide (1) overall advice and (2) concrete actions targeting weak areas.

Tone: professional and encouraging; highlight key insights; Focus on the most critical weaknesses and actionable steps.

Inputs:

- Student: ##{student}

- Test scores: pre-test ##{pre_score_text}, post-test ##{post_score_text}, change ##{score_delta_text}

- Test performance summary:

##{test_report}

- Dimension score overview:

##{score_overview}

- Assessment JSON:

##{evaluation_json}

Output JSON containing:

- general-advice-summary: overall advice summarizing performance and next priorities

- general-advice-recommended-resources: 1–2 must-read books/guidelines/cases or practice directions most relevant to this case

- detailed-advice: array (up to 3 items). Each item includes:

- dimension

- issue

- suggestion

**Human Expert Rating Rubric**

**Purpose and rating procedure**

This rubric is intended for independent human-expert rating of a specified learner from the complete de-identified PBL transcript. Rate five dimensions separately: Active Participation (AP), Clinical Reasoning (CR), Knowledge Accuracy (KA), Communication and Collaboration (CC), and Summarization and Reflection (SR). CR, KA, CC, and SR use the same constructs, evidence rules, and score anchors as the Evaluator Agent. AP is additionally rated by human experts because the Evaluator Agent scores only the other four dimensions. Assign one score per dimension on a 0-5 scale in 0.5-point increments and record a brief evidence-based justification.

**Shared scoring anchors**

0 = No observable evidence of the target competency in the learner's utterances.

1 = Minimal evidence; contributions are largely irrelevant, unsupported, or fundamentally inaccurate for the target dimension.

2 = Limited and fragmented evidence; major omissions, errors, or weak performance remain.

3 = Adequate but inconsistent evidence; performance is generally reasonable but has important gaps.

4 = Strong and consistent evidence; performance is clinically appropriate and discussion-relevant, with only minor omissions.

5 = Comprehensive, precise, and well-integrated evidence that substantively advances the discussion.

*Use a 0.5-point score only when performance falls clearly between two adjacent anchors. A score of 0 denotes absence of observable transcript evidence, not proof that the learner lacks the underlying ability.*

**Dimension-specific criteria**

Active Participation (AP): degree of sustained, relevant, and substantive engagement across the session, including initiating or re-engaging discussion, asking or answering questions, offering hypotheses or explanations, and contributing when learner input is expected. Consider both contribution frequency and substantive content. Do not award a high score for numerous superficial utterances, and do not penalize concise contributions when they are appropriately sustained and meaningful.

Clinical Reasoning (CR): integration of case cues; construction and refinement of a differential diagnosis; pathophysiologic explanation; evidence-based next-step investigation or management; and risk prioritization.

Knowledge Accuracy (KA): consistency with established medical knowledge; distinction between supported statements and over-inference; accuracy of numerical, drug, investigation, and complication details; and appropriate expression of uncertainty.

Communication and Collaboration (CC): direct and constructive response to peers or agents; clarity and professionalism; integration of others' ideas; and contribution to shared reasoning. In this study, this dimension concerns responsiveness within AI-mediated tutor/peer exchanges rather than real peer-to-peer teamwork. Do not score message frequency or length by itself.

Summarization and Reflection (SR): coverage of key conclusions across learning objectives; identification of uncertainty or unresolved issues; reflection on personal limitations; and a concrete improvement or self-study plan. Base this dimension primarily on evidence from the summary/reflection stage.

**Evidence and decision rules**

1. Score only the specified learner's observable utterances; use the group transcript only to interpret conversational context and opportunities to contribute.

2. Do not credit an idea stated only by another participant. Do not treat silence as a factual error or infer unobserved competence.

3. For CR and KA, compare the learner's claims with the case materials and accepted medical knowledge, serving the same reference function as the knowledge graph supplied to the Evaluator Agent. If the available reference is insufficient, note the limitation rather than infer accuracy.

4. Apply each dimension independently and avoid halo effects. AP concerns the extent and substance of engagement; CC concerns the quality of responsiveness, integration, and collaborative exchange. Do not lower CR, KA, or SR merely because the learner spoke infrequently.

5. Select the highest anchor for which the transcript provides sufficient evidence. Use a 0.5-point score only when the evidence justifies an intermediate judgment.

6. Experts should rate transcripts independently and remain blinded to other experts' ratings and automated scores until all ratings have been finalized.
