# Supplementary material for: Scaffolded AI-Supported Problem-Based Learning for Medical Interns: Exploratory Retrospectively Registered Randomized Controlled Evaluation With a Voluntary Feasibility Follow-Up
Source: JMIR Med Educ. 2026 Sep 3;12:e95039. doi: 10.2196/95039 (PMC13541230; doi:10.2196/95039)
Supplement: Supplementary file 4 [file mededu-v12-e95039-s003.docx]

# Blueprint of the pre- and post-test design.

Table S1 presents the test blueprint for our controlled study, mapping each scenario-specific learning objective (LO1–LO15) to parallel pre- and post-test items using a 1:2:2 design (two items per objective in each test). The blueprint aligns items with intended learning outcomes to support content validity and ensure comparable content coverage across the two tests.

Table S1. Combined objective-level assessment blueprint and item-to-objective mapping for the parallel pre- and post-tests (1:2:2 mapping). Each learning objective is assessed by two items in the pre-test and two parallel items in the post-test.

| **Scenario** | **Objective ID** | **Objective (short label)** | **Pre-test item IDs** | **Post-test item IDs** | **Domain / competency** | **Cognitive level (Bloom)** | **Difficulty target** |
| --- | --- | --- | --- | --- | --- | --- | --- |
| S1 | LO1 | Respiratory-source chest pain: key features & causes | PreQ01-PreQ02 | PostQ01-PostQ02 | Clinical reasoning / DDx | Apply/Analyze | Medium |
| S1 | LO2 | Lung "mass/space-occupying lesion": meaning & DDx | PreQ03-PreQ04 | PostQ03-PostQ04 | Diagnostic reasoning | Understand/Apply | Medium |
| S1 | LO3 | Workup for lung mass: imaging & pathology priorities | PreQ05-PreQ06 | PostQ05-PostQ06 | Diagnostic strategy / testing | Apply/Analyze | Medium |
| S1 | LO4 | Occupational/lifestyle risks beyond smoking (eg, diesel/PM) | PreQ07-PreQ08 | PostQ07-PostQ08 | Prevention / risk assessment | Understand/Apply | Medium |
| S2 | LO5 | Pulmonary nodule: CT features suggesting malignancy | PreQ09-PreQ10 | PostQ09-PostQ10 | Imaging interpretation | Apply/Analyze | Medium |
| S2 | LO6 | CT-guided lung biopsy: indications, limitations, risks | PreQ11-PreQ12 | PostQ11-PostQ12 | Procedures / patient safety | Understand/Apply | Medium |
| S2 | LO7 | Lung adenocarcinoma: pathology hallmarks & differentials | PreQ13-PreQ14 | PostQ13-PostQ14 | Pathology interpretation | Understand/Apply | Medium-Hard |
| S2 | LO8 | Molecular testing (EGFR/ALK/ROS1): clinical significance | PreQ15-PreQ16 | PostQ15-PostQ16 | Precision medicine | Understand/Apply | Hard |
| S3 | LO9 | Early NSCLC surgery: indications, approach, perioperative principles | PreQ17-PreQ18 | PostQ17-PostQ18 | Treatment decision-making | Apply/Analyze | Medium |
| S3 | LO10 | MDT role in individualized early lung cancer planning | PreQ19-PreQ20 | PostQ19-PostQ20 | Team-based care / SDM | Understand/Apply | Medium |
| S3 | LO11 | Non-surgical options (SBRT/RFA): indications & comparison | PreQ21-PreQ22 | PostQ21-PostQ22 | Treatment alternatives | Apply/Analyze | Medium-Hard |
| S3 | LO12 | Adjuvant therapy after resection: indications & evidence | PreQ23-PreQ24 | PostQ23-PostQ24 | Evidence-based oncology | Analyze/Evaluate | Hard |
| S4 | LO13 | Perioperative psychological responses & impact on recovery | PreQ25-PreQ26 | PostQ25-PostQ26 | Psychosocial care | Understand/Apply | Medium |
| S4 | LO14 | Patient-centered communication (empathy, teach-back, empowerment) | PreQ27-PreQ28 | PostQ27-PostQ28 | Communication / SDM | Apply/Analyze | Medium |
| S4 | LO15 | Integrated support: psych, rehab, vocational, peer support | PreQ29-PreQ30 | PostQ29-PostQ30 | Care coordination / rehab | Apply/Evaluate | Medium-Hard |

**Example blueprint-aligned items for S1-LO1 (PreQ01-PreQ02; PostQ01-PostQ02)**

**Scenario (S1):** Chest pain (respiratory system differential diagnosis)

**Objective ID:** LO1

**Objective (short label):** Respiratory-source chest pain: key features & causes

**Domain / competency:** Clinical reasoning / differential diagnosis

**Cognitive level (Bloom):** Apply/Analyze

**Difficulty target:** Medium

**PreQ01 (single-best-answer multiple-choice question).**

**Question:** Right-sided chest pain that worsens with inspiration and coughing, with a pleural friction rub heard on auscultation, is most likely caused by:

A. Dry (fibrinous) pleuritis

B. Stable angina pectoris

C. Gastroesophageal reflux disease

D. Aortic dissection

E. Prodromal stage of herpes zoster

*Correct answer: A*

**PreQ02 (single-best-answer multiple-choice question).**

**Question:** Right-sided chest pain with low-grade fever and cough, with localized dullness to percussion and decreased vocal fremitus, is most likely caused by:

A. Small pleural effusion or pleuritis

B. Stable angina pectoris

C. Gastroesophageal reflux disease

D. Chest wall muscle strain

E. Prodromal stage of herpes zoster

*Correct answer: A*

**PostQ01 (single-best-answer multiple-choice question).**

**Question:** Which of the following features more strongly suggests that chest pain originates from the respiratory system?

A. Stabbing/knife-like pain that worsens with deep inspiration or coughing

B. Exertional crushing pain radiating to the left upper limb

C. Retrosternal burning pain with acid regurgitation, worse when supine

D. Sudden tearing pain radiating to the back

E. Chest pain relieved by lying supine at night

*Correct answer: A*

**PostQ02 (single-best-answer multiple-choice question).**

**Question:** Which of the following findings more strongly suggests that chest pain is caused by pulmonary embolism?

A. Exertional retrosternal crushing pain relieved by nitroglycerin

B. Burning pain after meals with acid regurgitation, relieved by antacids

C. Sudden chest pain with dyspnea, hypoxemia, and signs of DVT

D. Dull pain relieved by deep inspiration

E. Chest wall pain with clearly localized tenderness

*Correct answer: C*

Supplementary item-level psychometric analyses

To supplement the blueprint-based content validity evidence, we conducted post hoc item-level psychometric analyses using actual participant responses from the randomized educational evaluation. We also invited three domain experts to independently rate the difficulty of all pre- and post-test items on a 5-point scale; inter-rater agreement was summarized using Kendall’s W.

Item-level psychometric statistics were as follows: for the pre-test form, KR-20=0.7092, mean item difficulty=0.7827, and mean point-biserial discrimination=0.2799; for the post-test form, KR-20=0.7331, mean item difficulty=0.8840, and mean point-biserial discrimination=0.3951.

Expert-rated item difficulty and inter-rater agreement were as follows: overall Kendall’s W=0.867, pre-test Kendall’s W=0.9004, and post-test Kendall’s W=0.8423. The mean expert difficulty ratings were 3.3778 for the pre-test form and 3.4111 for the post-test form.

These results indicate acceptable internal consistency and item discrimination for both forms. Expert-rated difficulty was highly consistent across raters and similar between the pre-test and post-test forms, supporting blueprint-level comparability of item difficulty. Formal parallel-forms reliability was not estimated because the forms were administered in a fixed pre/post sequence without counterbalancing or anchor items.
