# Supplementary material for: Scaffolded AI-Supported Problem-Based Learning for Medical Interns: Exploratory Retrospectively Registered Randomized Controlled Evaluation With a Voluntary Feasibility Follow-Up
Source: JMIR Med Educ. 2026 Sep 3;12:e95039. doi: 10.2196/95039 (PMC13541230; doi:10.2196/95039)
Supplement: Supplementary file 5 [file mededu-v12-e95039-s005.docx]

# **Descriptive Categorization of Open-Ended Feedback**

Open-ended feedback was used as descriptive illustration rather than formal qualitative thematic analysis. Feedback entries could be assigned to more than one descriptive category; therefore, category counts are not mutually exclusive. Feedback IDs are de-identified.

## Category Summary

| Descriptive category | Unique feedback entries |
| --- | --- |
| Structured clinical reasoning support | 5 |
| Perceived learning gain | 4 |
| Reduced pressure and freer expression | 4 |
| Content coverage and case realism | 3 |
| Interaction depth, flow, or tutor questioning | 3 |
| Assessment or questionnaire design | 2 |
| Content repetition, overlap, or concision | 2 |
| Interface usability and navigation | 2 |
| Conversational accuracy or AI misunderstanding | 1 |
| Learning tool and review support | 1 |
| Time burden or session control | 1 |

## Complete Categorized Feedback

| Feedback ID | Valence | Descriptive category | Original comment | English translation |
| --- | --- | --- | --- | --- |
| F01 | mixed | Content coverage and case realism; Structured clinical reasoning support; Reduced pressure and freer expression; Conversational accuracy or AI misunderstanding | 涵盖知识及内容十分全面完善，选取病例也有代表性。但在课堂上的发言内容ai并不能完全理解甚至误解，会有些懊恼。总的来说是一次十分好的体验，回想起当年在PBL课堂上羞于举手的感觉，在这里可以畅所欲言并系统的学习到知识，十分感谢！ | The system covered comprehensive content and representative cases, supported freer expression than classroom PBL, and helped systematic learning, but the AI sometimes misunderstood the learner's comments. |
| F02 | improvement | Assessment or questionnaire design | 前测题目难度可以有所提高。 | The pre-test items could be made more difficult. |
| F03 | positive | Reduced pressure and freer expression | 学习过程很有代入感 | The learning process felt immersive. |
| F04 | improvement | Interaction depth, flow, or tutor questioning | 互动感可以再加强，可以增加一点讨论间的教师反问。 | The interaction could be strengthened, for example by adding more teacher counter-questioning during discussion. |
| F05 | positive | Content coverage and case realism | 案例选的很恰当，很贴合实际，很详细。 | The case selection was appropriate, realistic, detailed, and closely connected to practice. |
| F06 | improvement | Assessment or questionnaire design | 调查问卷可以再多几个问题。 | The survey questionnaire could include more questions. |
| F07 | mixed | Content coverage and case realism; Content repetition, overlap, or concision | 很全面，受益匪浅，但有重复内容，感觉精简一下会更好 | The content was comprehensive and beneficial, but some repeated content could be streamlined. |
| F08 | improvement | Time burden or session control | 好像不能中途暂停，有点费时 | The session seemed difficult to pause midway and felt somewhat time-consuming. |
| F09 | positive | Structured clinical reasoning support; Perceived learning gain | 这个学习模式最重要的是，让我学会了系统的临床思维模式！太好了 | The learning mode helped the learner develop a systematic clinical reasoning pattern. |
| F10 | improvement | Interaction depth, flow, or tutor questioning | 该案例学习的讨论进程很快。 | The discussion process in this case moved quickly. |
| F11 | improvement | Interface usability and navigation | 我的发言怎么换行呢？只能挤在一段写吗？想按”enter“换行结果是发送 | The learner had difficulty entering line breaks because pressing Enter sent the message. |
| F12 | positive | Structured clinical reasoning support; Reduced pressure and freer expression; Perceived learning gain | 整体体验很好，互动式学习让我更愿意主动思考和表达，完成学习后感觉对相关知识点掌握得更扎实，也更有信心应用到实际问题中。 | The overall experience was good; interactive learning encouraged active thinking and expression, strengthened knowledge mastery, and increased confidence in applying knowledge to practical problems. |
| F13 | positive | Structured clinical reasoning support | 平台的学习流程比较清晰，对诊断和处理思路的训练很有帮助。 | The platform's learning workflow was clear and helpful for training diagnostic and management reasoning. |
| F14 | improvement | Content repetition, overlap, or concision | 场景123的一些学习目标有重合 | Some learning objectives across scenarios 1 to 3 overlapped. |
| F15 | improvement | Interaction depth, flow, or tutor questioning; Interface usability and navigation; Learning tool and review support | 流程引导可优化：当前“请题1、请题2”的系统提示稍显机械，建议改为更自然的引导语，或设置“点击展开”的折叠式任务栏，减少界面干扰。 讨论体验增强：在实时讨论中，若能支持快速引用他人观点并回复，或增加临时标注/高亮功能，将有助于提升讨论的针对性与连贯性。 学习工具整合：右侧讨论记录区域若能支持关键词筛选或按学生角色筛选，将更方便回顾与总结。另外，可考虑嵌入简易思维导图工具，辅助梳理诊断逻辑。 | The learner suggested more natural guidance prompts, collapsible task panels, tools for quoting or highlighting comments, filtering discussion records, and mind-map support. |
| F16 | positive | Structured clinical reasoning support; Reduced pressure and freer expression | 这种模拟PBL的形式比传统课堂更灵活，既能按照自己的节奏思考，又能通过系统引导逐步完善诊疗思路 | This simulated PBL format was more flexible than a traditional classroom, allowing self-paced thinking while system guidance progressively refined diagnostic and treatment reasoning. |
| F17 | positive | Perceived learning gain | 怎么说呢，这个pbl啊，只要稍微认真做一下，分数进步就能很大，我也是惊呆，效果这么明显么? | The learner was surprised by the magnitude of score improvement after serious engagement with the PBL activity. |
| F18 | positive | Perceived learning gain | 感觉比单纯看书更容易理解和记忆。 | The learner found it easier to understand and remember than reading alone. |
